# Inactivation times from 290 to 315 nm UVB in sunlight for SARS coronaviruses CoV and CoV-2 using OMI satellite data for the sunlit Earth

**DOI:** 10.1007/s11869-020-00927-2

**Published:** 2020-09-15

**Authors:** Jay Herman, Bryan Biegel, Liang Huang

**Affiliations:** 1 University of Maryland Baltimore County JCET, Baltimore, MD USA; 2 NASA Ames Research Center, Moffett Field, CA USA; 3 Science Systems and Applications, Lanham, MD USA

**Keywords:** COVID-19, SARS CoV, Inactivation, Radiative transfer, Ozone, UVB, UVC

## Abstract

UVB in sunlight, 290–315 nm, can inactivate SARS CoV and SARS CoV-2 viruses on surfaces and in the air. Laboratory exposure to ultraviolet irradiance in the UVC range inactivates many viruses and bacteria in times less than 30 min. Estimated UVB inactivation doses from sunlight in J/m^2^ are obtained from UVC measurements and radiative transfer calculations, weighted by a virus inactivation action spectrum, using OMI satellite atmospheric data for ozone, clouds, and aerosols. For SARS CoV, using an assumed UVC dose near the mid-range of measured values, *D*_90_ = 40 J/m^2^, 90% inactivation times *T*_90_ are estimated for exposure to midday 10:00–14:00 direct plus diffuse sunlight and for nearby locations in the shade (diffuse UVB only). For the assumed *D*_90_ = 40 J/m^2^ model applicable to SARS CoV viruses, calculated estimates show that near noon 11:00–13:00 clear-sky direct sunlight gives values of *T*_90_ < 90 min for mid-latitude sites between March and September and less than 60 min for many equatorial sites for 12 months of the year. Recent direct measurements of UVB sunlight inactivation of the SARS CoV-2 virus that causes COVID-19 show shorter *T*_90_ inactivation times less than 10 min depending on latitude, season, and hour. The equivalent UVC 254 nm *D*_90_ dose for SARS CoV-2 is estimated as 3.2 ± 0.7 J/m^2^ for viruses on a steel mesh surface and 6.5 ± 1.4 J/m^2^ for viruses in a growth medium. For SARS CoV-2 clear-sky *T*_90_ on a surface ranges from 4 min in the equatorial zone to less than 30 min in a geographic area forming a near circle with solar zenith angle < 60^O^ centered on the subsolar point for local solar times from 09:00 to 15:00 h.

**Electronic supplementary material:**

The online version of this article (10.1007/s11869-020-00927-2) contains supplementary material, which is available to authorized users.

## Introduction

Onset of coronavirus induced diseases (e.g., 2002, severe acute respiratory syndrome, SARS, (virus, SARS-CoV), 10 years later Middle East respiratory syndrome MERS (virus, MERS-CoV), and starting in 2019, a new viral mutation, SARS-CoV-2, causing COVID-19, has promoted increased interest in methods of deactivating the virus on surfaces through chemical biocidal agents (Kampf et al. 2020) or UVGI (ultraviolet germicidal irradiation) (Anderson et al. 2013; Bedell et al. 2016; Heßling et al. 2020; Lytle and Sagripanti 2005; Sagripanti and Lytle 2020; Kowalski et al. 2009; Kowalski 2009). Most of the work on effective UVGI was performed with radiation in the UV-C range (100–280 nm), usually from low pressure mercury lamps at 254 nm. Sunlight reaching the Earth’s surface does not contain significant irradiance for wavelengths less than 290 nm because of absorption by atmospheric ozone and increased Rayleigh scattering with decreasing wavelength. However, there is smaller but significant viral inactivation by UVB wavelengths contained in sunlight in the range 290 to 315 nm (Eisenstark 1987; Nelson et al. 2018). Recently, it has been shown directly that UVB in amounts present in summer sunlight can inactivate the SARS-CoV-2 viruses efficiently (Ratnesar-Shumate et al. 2020) when the virus droplets are dried onto stainless-steel mesh (90% in about 10 min) and in growth medium in about 17 min.

Inactivation sensitivity to 254 nm UVC radiation is frequently measured in terms of the dose *D*_90_ (J/m_2_) needed to reduce the number of active virus particles by 90%. Measurements by Walker and Ko (2007) showed an inactivation of 87.8% aerosolized murine hepatitis coronavirus (MHV) for an exposure to 254 nm UVC of 5.99 J/m^2^, which corresponds to *D*_90_ = 6.6 J/m^2^ (Table 1 and Online Resource 1: Figs. S1 to S4). This inactivation dose is similar to that of the Berne virus Coronaviridae (Weiss and Horzinek 1986; Lytle and Sagripanti 2005; Kowalski et al. 2020a, b), which we estimate to be 7.1 J/m^2^ (Fig. S2). A measurement by Liu et al. 2003 on the MHV coronavirus in liquid yielded *D*_90_ = 95 J/m^2^ (Fig. S4). While both viruses are in the coronavirus family, their UV inactivation sensitivity may not be representative of either the SARS-CoV or MERS-CoV variants. Inactivation of CoV-P9 by UVC (Duan et al. 2003) showed undetectable amounts of virus after 60 min of irradiation to 0.9 W/m^2^, which, in a company report, Kowalski et al. (2020a, b) estimated D_90_ = 40 J/m^2^. Evaluation of a laboratory study by Kariwa et al. (2004) on SARS CoV (Hanoi) gives 46 J/m^2^ (Fig. S1) and SARS CoV (Urbani) 1826 J/m^2^ (Fig. S3) based on laboratory studies by Darnell et al. (2004). Kowalski et al. (2020a, b) obtained different values of *D*_90_ = 134 J/m^2^ and 2410 J/m^2^, respectively. https://avdc.gsfc.nasa.gov/pub/DSCOVR/JayHerman/COVID-19/).

**Table 1:** Estimation of UVC D90 (Figs. S1 to S4, items 1-6) and later section for items 7–8

Item	*D*_90_ (J/m^2^)	Data reference
1	7.1	Berne-CV (Weiss and Horzinek 1986).
2	40	CoV-P9 by UVC (Duan et al. 2003; Kowalski et al. 2020a, b)
3	46	SARS CoV (Hanoi) (Kariwa et al. 2004)
4	1826	SARS-CoV (Urbani) (Darnell et al. 2004)
5	95	Murine Hepatitis Coronavirus (MHV) (Liu et al. 2003)
6	6.6	Murine Hepatitis Coronavirus (MHV) Airborne (Walker and Ko 2007)
7	3.2	SARS CoV-2 dried on steel mesh UVC *D*_90_ estimated
8	6.5	SARS CoV-2 in growth medium UVC *D*_90_ estimated

A fast calculation method is used (Online Resource 2: Eqns. S2 to S10) for globally estimating the inactivation of SARS CoV by sunlight using satellite data, the action spectrum from Lytle and Sagripanti (2005), and a nominal value of *D*_90_ = 40 J/m^2^ (close to the SARS CoV Hanoi value of 46 J/m^2^) to estimate the time for 90% inactivation *T*_90_ for a large number of cities worldwide (Online Resource 3 Table S1) and the number of months where monthly averages (1 to 12) of *T*_90_ ≤ 120 min. The *T*_90_ results are linearly proportional to the assumed value of *D*_90_.

Heßling et al. (2020) discuss possible reasons for the large variations in measured *D*_90_, items 1 to 6 in Table 1. They also conclude, “The calculated upper limit for the log-reduction median dose (in low-absorbance media) is 10.6 mJ/cm^2^, but the probably more precise estimation is 3.7 mJ/cm^2^.” This corresponds to 106 J/m^2^ and 37 J/m^2^, respectively, the latter close to the nominal value of 40 J/m^2^ assumed here.

Since the Ratnesar-Shumate et al. (2020), (RS) measurements are made using simulated clear-sky sunlight in the 290 to 315 nm range, the fast calculation method based on *A*(*λ*) for measurements made at 254 nm is not needed for estimating *T*_90_ as a function of solar UVB irradiance for their measurement conditions. In order to generalize the RS measurements, an estimate of the equivalent 254 nm *D*_90_ amount is obtained by matching their measurement conditions and results using the TUV radiative transfer calculations. The estimated 254 nm inactivation *D*_90_ of SARS CoV-2 (Table 1) gives globally distributed estimates of RS *T*_90_.

## Inactivation calculation by UVGI from sunlight UVB

**Fig. 1:**
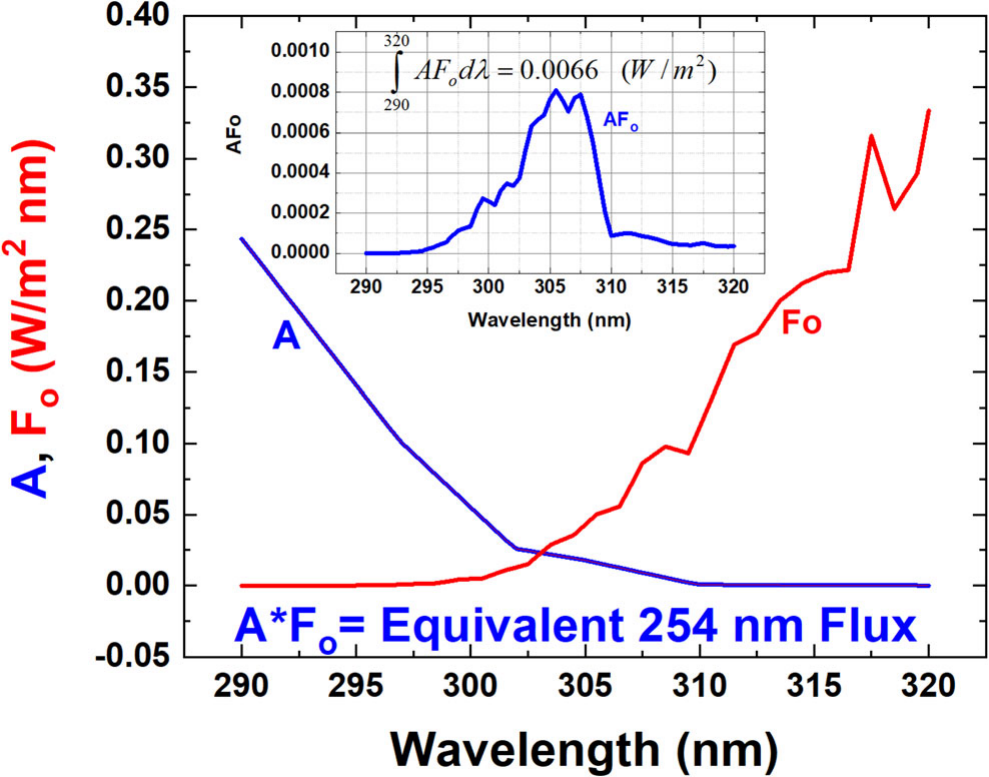
The viral inactivation spectrum *A*(*λ*) normalized to 1 at 254 nm (blue), solar irradiance (red) at the Earth’s surface *F*_O_(W/m^2^ nm), and the product AF_O_(*λ*) (inset) for *θ* = 30^O^ and *Ω* = 325 DU

To estimate the effect of sunlight in the 290 ≤ *λ* ≤ 315 nm UVB range that reaches the Earth’s surface at significant intensity, a transfer function from 254 nm to UVB (290–315 nm), or action spectrum *A*(*λ*) is needed that is normalized to 1 at 254 nm (Fig. 1 from Lytle and Sagripanti 2005) (Eqs. 1 and 2 and Fig. 1). The analysis is based on an application of TUV (Madronich 1993, 1995) atmospheric radiative transfer calculation using ozone monitoring instrument (OMI) satellite data total column ozone (TCO_3_), estimated cloud transmission *C*_*T*_, and absorbing aerosol transmission *C*_*A*_ to derive a useful formulation for high-speed evaluation (Herman 2010; Herman et al. 2018, 2020). The results are presented in terms of dosage (*D*) in J/m^2^ and inactivation time (*T*_90_) in minutes from UV solar radiation to achieve 90% inactivation relative to the *D*_90_ exposure at 254 nm.

For SARS CoV, estimates of UVB *T*_90_ are obtained from UVC measurements for 4 open land sites and 190 cities in Europe, North America, South America, Asia, and Australia. Analysis is presented for monthly averages <*T*_90_> of clear and cloudy days from 12:00 ± 4 h local solar time and compared to noon virus inactivation times. The goal is to determine how many days of the year the inactivation time from UVB sunlight was short enough to have a significant impact on decontamination of surfaces and airborne coronaviruses in direct sunlight or in nearby shade. Most of the analysis is based on the assumption that laboratory inactivation data for virus particles suspended in an aqueous solution, which absorbs some of the UVC, applies to surfaces and aerosolized particles suspended in air and that *A*(*λ*) is valid for coronaviruses. The data in Table 1 suggests that *T*_90_ for aerosolized virus particle in air or on surfaces is shorter than viruses in liquids. The *T*_90_ times for both SARS CoV and SARS CoV-2 on surfaces are given as a function of latitude and season using a uniform calculation method for all cases considered.

Key components for estimating *T*_90_ for a coronavirus are (1) an estimate of the normalized action spectra *A*(*λ*) representing the relative efficiency for a wavelength *λ* compared to the much stronger inactivation rate at the UVC wavelength 254 nm; (2) a calculated estimate of the solar irradiance reaching the Earth’s surface as a function of solar zenith angle (*θ* = SZA), total column ozone amount (*Ω* = TCO_3_) over a specified site, fractional cloud transmission *C*_*T*_ of UV irradiance using measured Lambert equivalent reflectivity (LER) of the scene, and fractional absorbing aerosol transmission (*C*_*A*_), all as a function of latitude *ζ*, longitude *ϕ*, altitude *z*, and day of the year (DOY). The same method is applied for SARS CoV-2 after calculating the equivalent 254 nm *D*_90_.

Atmospheric data is obtained from measurements by the OMI onboard the US Aura satellite (2004–present). OMI is a polar orbiting nadir and side viewing satellite instrument (2600-km-wide swath on the surface) providing near global coverage (nadir resolution field of view 13 km × 24 km) once per day from a 90-min polar orbit with an equator crossing time of approximately 13:30 local solar time (LST) (Levelt et al. 2018). For computational purposes, the input data have been averaged onto a 1^O^ × 1^O^ latitude × longitude grid https://avdc.gsfc.nasa.gov/pub/tmp/OMI_Daily_O3_and_LER/. OMI data are filtered to remove data from bad detector pixels and for the known so-called row anomaly (Schenkeveld et al. 2017).

Additional data from the Deep Space Climate Observatory (DSCOVR) Earth Polychromatic Imaging Camera (EPIC) in orbit about the Earth-Sun Lagrange-1 point L_1_, 1.5 million kilometers from Earth, are used for science products and color pictures of the entire sunlit Earth at specific Greenwich Mean Times (GMT).

The solar irradiance spectrum output (W/m^2^ nm) at the Earth’s surface *F*(*ζ*,*ϕ*,*θ*,*λ*,*Ω*,*z*), calculated from the TUV model, is obtained for 100 ≤ *Ω* ≤ 600 Dobson Units (1 DU = 2.687 × 10^16^ molecules/cm^2^), for SZA range 0^O^ ≤ *θ* ≤ 80^O^, and for an altitude range 0 ≤ *z* ≤ 8 km. The TUV spectral output is multiplied by a normalized action spectrum *A*(*λ*) and integrated over the non-zero range of *F*(*λ*)*A*(*λ*) (Fig. 1 and Eq. 1).

1 $$ P\left(\varsigma, \phi, \theta, \Omega, z\right)=\underset{290}{\overset{320}{\int }}F\left(\varsigma, \phi, \theta, \lambda, \Omega, z\right)A\left(\lambda \right) d\lambda $$

The TUV output *P*(*θ*,*Ω*) can be used as a table look up or converted to a very accurate functional fit (Online Resource 2: Eqns. S2 to S10), where the latter is much faster for computational purposes. The small error estimates for this type of functional fit are also given (Herman 2010).

The action spectrum *A*(*λ*) (Lytle and Sagripanti 2005) is approximated by a rational fraction fit for the range 290 ≤ *λ* ≤ 320 nm (Eq. 2 and Table 2):

2 $$ A\left(\lambda \right)=\frac{a_1+{a}_2{\theta}^{0.5}+{a}_3\theta }{1+{b}_1{\theta}^{0.5}+{b}_2\theta } $$

**Table 2:** Coefficients for A(*λ*) and see Fig. 1

*n*	*a*_*n*_	*b*_*n*_
1	0.03185621255581713	− 0.1171817797253023
2	− 0.003565593470829207	0.003433857833125471
3	9.976358190920908x10^-05^	

For a case at *z* = 0 km, *F*_*O*_ = *F*(*ζ*,*ϕ*,*θ*,*λ*,*Ω*,*0*), *Ω* = 325 DU, and *θ* = 30^O^. *P*(*ζ*,*ϕ*,*θ*,*Ω*,*0*) = 0.0066 ± 0.0013 W/m^2^ as shown in Fig. 1. The 20% uncertainty in *P*, ± 0.0013, arises from uncorrelated error estimates for *A*(*λ*). Figure 1 shows clear-sky irradiance at the ground *F*_*O*_*A*(*λ*) peaking near 305 nm. While the amount of clear-sky 305 nm solar irradiance at the ground is small compared to longer wavelengths, because of attenuation by ozone and Rayleigh scattering, it is significant for viral inactivation (Fig. 1)

The next step is translating the atmospheric calculations of *P*(*θ*,*Ω*,*0*) for the Earth’s surface at sea level (*z* = 0) into a 90% inactivation time *T*_90_ as a function of SZA. Laboratory values of the logarithmic decay of viruses of starting number *N*_*O*_ exposed to 254 nm UV light for exposure *D* (J/m^2^) are measured as a function of time to determine the slope k of the decay curve as a function of the survival fraction N/N_O_ (Eq. 3).

3 $$ N/{N}_O={e}^{- kD}\kern0.5em or\kern0.5em k=-\frac{1}{D} Ln\left(N/{N}_O\right) $$

When *D* = *D*_90_ (J/m^2^), it is an exposure representing 10% survival, *N*/*N*_*O*_ = 0.1. More complicated two-slope inactivation models have been used when the inactivation vs. *D* show two log-linear slopes (Kowalski et al. 2020a, b) for inactivation times greater than *T*_90_.

**Fig. 2:**
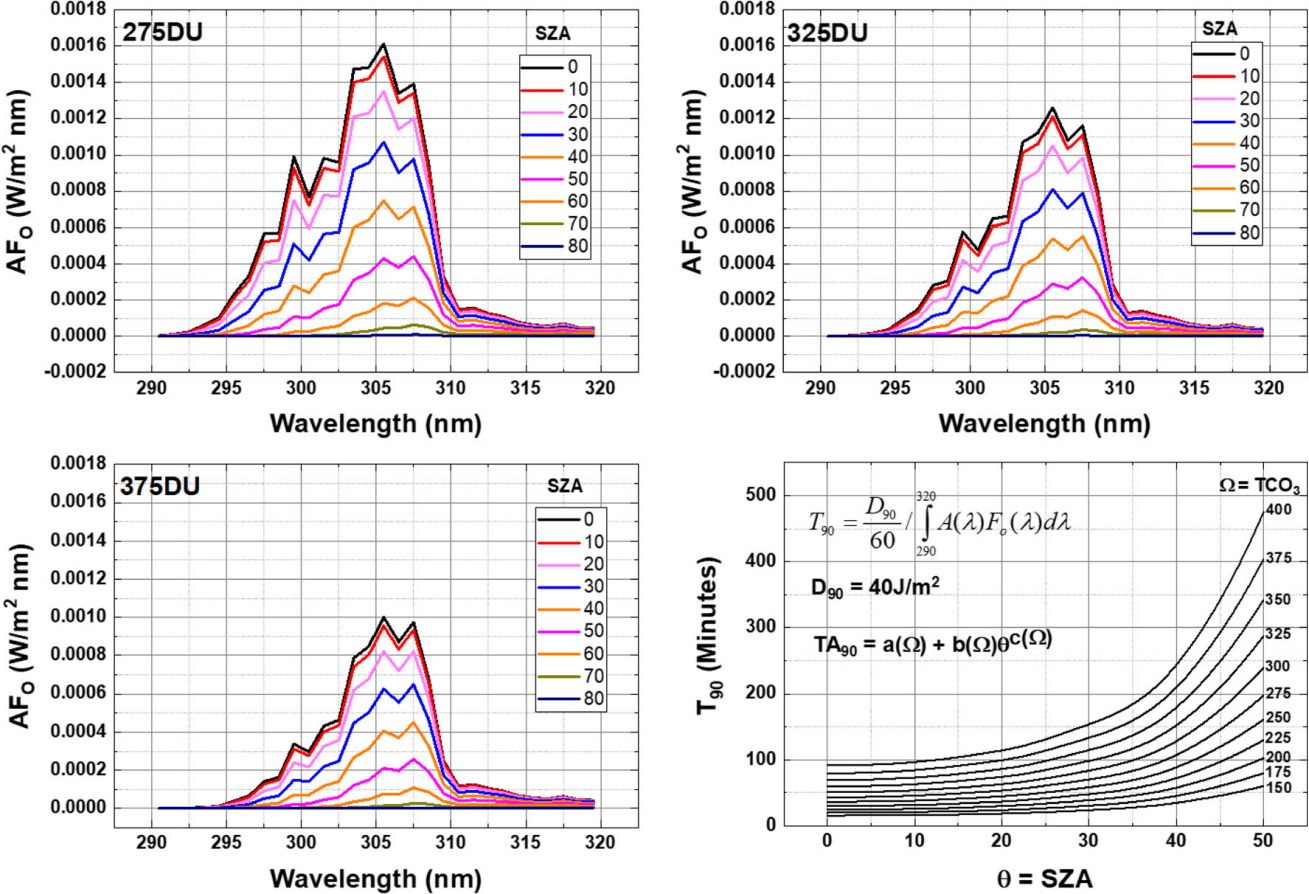
A sample calculation of AFO for clear-sky conditions for *Ω* = 150 - 400 DU, and the 90% inactivation time T_90_ as a function of SZA using Eqs. 1, 2, and 4. TA_90_ is a polynomial approximate fit to T_90_

Figure 2 expands the calculation for a range of SZA and TCO_3_ values from 150 to 400 DU, spanning the range of TCO_3_ expected over latitudes from 65^O^S to 65^O^N on most days of the year. Average equatorial TCO_3_ is smaller than average mid-latitude values and the minimum SZA is smaller so that equatorial *T*_90_ is shorter than for mid-latitudes.

To obtain the 90% inactivation time *T*_90_ from the integrated product of *A*(*λ*)*F*_O_(*λ*) and the measured value of *D*_90_, the following formula (Eq. 4) is used:

4 $$ {T}_{90}=\frac{D_{90}}{60}/\underset{290}{\overset{320}{\int }}A\left(\lambda \right){F}_o\left(\lambda \right) d\lambda $$

The factor 60 converts the units from seconds to minutes. For SARS CoV, the nominal value *D*_90_ = 40 J/m^2^ is used in the graphs below as a middle value in Table 1 close to the SARS CoV Hanoi value of 46 J/m^2^.

**Fig. 3:**
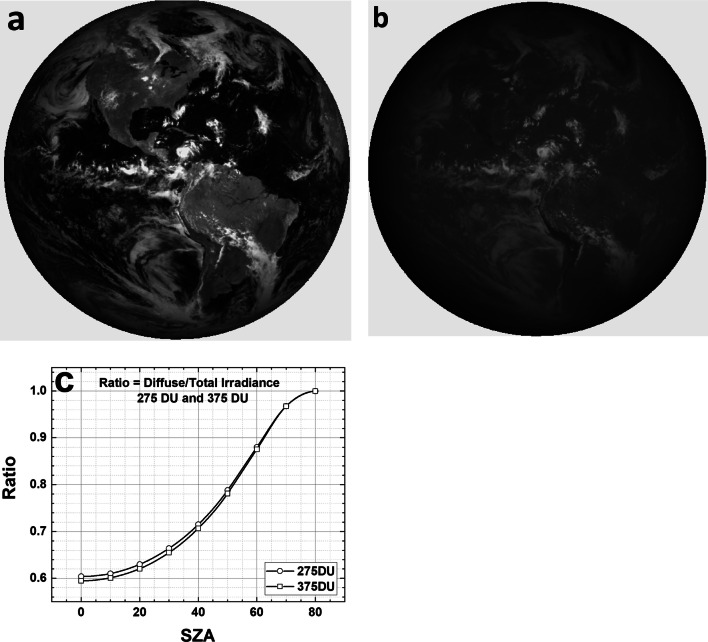
**a** The Earth as viewed by DSCOVR EPIC at 317 nm showing the effect of Rayleigh scattering. **b** the same scene but at 780 nm. **c** the calculated ratio of *P*(*λ*) diffuse divided by diffuse plus direct sunlight as a function of SZA for TCO_3_ = 275 DU and 375 DU

For UV irradiance in the vicinity of 305 nm, there is considerable scattered diffuse light caused by strong Rayleigh scattering causing a clear atmosphere in longer wavelengths (Fig. 3a) to appear more like a light fog in short wavelength UVB (Fig. 3b). Surfaces that appear to be in the shade in visible light are bathed in diffuse light (Fig. 3) that is 60% to 70% of the total *A*(*λ*) weighted irradiance (diffuse + direct) for *θ* < 40^O^ (Fig. 3). *T*_90_(diffuse, *θ* < 40^Ο^) is 1.4 to 1.7 times *T*_90_(total, *θ* < 40^Ο^). Figures 2 and 3 imply that horizontal surfaces permanently left outside and exposed to midday solar UVB irradiances will have coronaviruses 90% inactivated in less than 120 min for mid- or low-latitude sites for *D*_90_ = 40 J/m^2^. For the UVB inactivation measurements on SARS CoV-2 (Ratnesar-Shumate et al. 2020), *T*_90_ values in Fig. 2 are reduced by a factor of 12.5 (for SZA = 0^o^, 6 min TCO_3_ = 375DU, 5 min TCO_3_ = 325 DU, 3.5 min TCO_3_ = 275 DU) .

For airborne virus particles the actinic flux or fluence is of interest, which is the sum of the upward and downward irradiance, increasing the total UVB exposure by about 5% near the surface of the Earth relative to the total downward irradiance. There is evidence that aerosolized airborne virus particles are more susceptible to UVC than samples measured in liquid (Table 1).

## Estimation of *T*_90_ from satellite data

**Fig 4:**
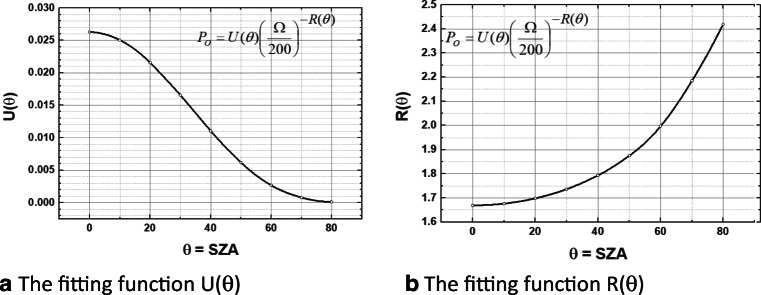
**a** The fitting function *U*(*θ*) in Eq. 5. **b** The fitting function *R*(*θ*) in Eq. 5

To efficiently expand the calculation of *T*_90_ to use satellite data on a global basis for daily calculations as a function of latitude and longitude, an efficient representation (Eq. 5) is needed for the calculated irradiances from the TUV radiative transfer calculation over the *θ* × *Ω* × *z* space for the Earth at 1 astronomical unit AU distance from the sun. An allometric form (Eq. 5) accurately fits the TUV output from (Eq .1) for a wide range of *θ* and *Ω* (Herman 2010), and altitude *z*, where *U*(*θ*) and *R*(*θ*) are numerical fitting coefficients defined below (Online Resource 2 and Fig. 4). *R*(*θ*) is an improved version of the radiation amplification factor.

5 $$ P\left(\theta, \Omega, z,{C}_T{C}_A,H,{D}_S\right)=U\left(\theta \right){\left(\frac{\Omega}{200}\right)}^{-R\left(\theta \right)}{C}_T{C}_AH(z){D}_S $$

$$ \frac{\partial P}{P}=-R\left(\theta \right)\frac{\mathrm{\partial \Omega }}{\Omega} $$

Here, the dependence on latitude and longitude (*ζ*, *ϕ*) is not explicitly indicated:

*C*_*T*_: cloud transmission fraction between 0 and 1
*C*_*A*_: aerosol transmission fraction between 0 and 1
*H*(*z*): topography height factor relative to sea level between 0 and 8 km
*D*_*S*_: correction factor for the distance of the Earth from the sun relative to 1 AU

These quantities are quantitively defined (Online Resource 2: Eqns. S2 to S10, Herman and Celarier 1997; Mok et al. 2018; Torres et al. 2007).

The principal source of error in the radiative transfer calculation method is from uncertainty in the measured ozone value of Δ*Ω* = ± 1 to ± 2%, which would cause an error in in *P*_O_(*θ*,*Ω*) of 1.7Δ*Ω* for low *θ* and 2.4Δ*Ω* for *θ* = 80^O^ (Eq 5, Online Resource 2: Fig. S5). Highly polluted cities will have less irradiance at the ground than estimated from Eq. 5 because of the area averaging over the satellite field of view that can include less polluted areas. These errors have been shown to be about 20% calculated overestimation of irradiance at the surface (Lakkala et al. 2020). The accuracy of the TUV calculation compared to ground-based measurements for clear skies has been evaluated (Michalsky and Kiedron 2008) showing an irradiance overestimation for TUV of 1 to 2%. The minimum inactivation times central to this analysis are for days with low reflectivity (little or no clouds) and low aerosol absorption. Errors in the fitting functions are negligible (Herman 2010) by comparison.

Applying the radiative transfer fitting equations to the nearly complete global coverage afforded by OMI’s field of view enables *P*(*λ*,*θ*,*ζ*, *ϕ*,*z*) to be determined for any location (*ζ*, *ϕ*) as a function of DOY (January 2005 to December 2019). Only the 2019 data are shown. *T*_90_ is shown for 12 locations representing major cities in the USA, Asia, Australia, Europe, and South America, some strongly affected by COVID-19. A total of 190 cities and 4 land sites are listed (Online Resource 3: Table) showing the 2019 minimum of 12 noontime monthly averages <*T*_90_> of daily noontime *T*_90_ (_Min_ <*T*_90_> _12:00_, columns 4 and 6), and the number of months *N*_*m*_ (columns 5 and 7 ) in 2019 where noontime <*T*_90_> is 120 min or less**.** These averages include the effects of daily cloud and aerosol cover.

**Fig. 5:**
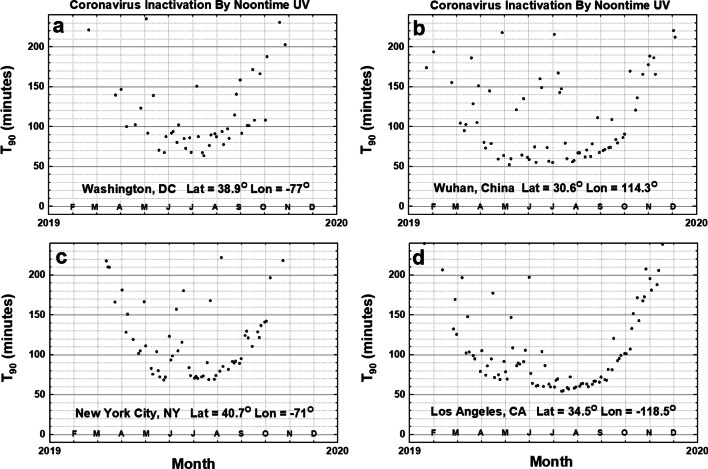
Noontime *T*_90_ inactivation times for coronavirus in minutes for Washington DC, US; Wuhan, CN; New York City, US; and Los Angeles, US; and using *D*_90_ = 40 J/m^2^. For the calculated Ratnesar-Shumate et al. (2020) SARS CoV-2 *D*_90_ = 3.2 J/m^2^, *T*_90_ in June would be about 4.8 for Washington, 4.4 for Wuhan, 5.6 New York City, and 4.8 min for Los Angeles. Figures are truncated at 240 min

For cities at mid-latitudes between 30^O^ and 40^O^ (Fig. 5), there is a period where the noontime *T*_90_ is approximately 60–80 min and lasts for several months. For New York City at 40.7^O^ N, the number of months, *T*_90_ < 80 min, is less (May to August) than for Los Angeles (April to September). The scatter in the points is mainly due to clouds causing time-varying *C*_*T*_.

**Fig. 6:**
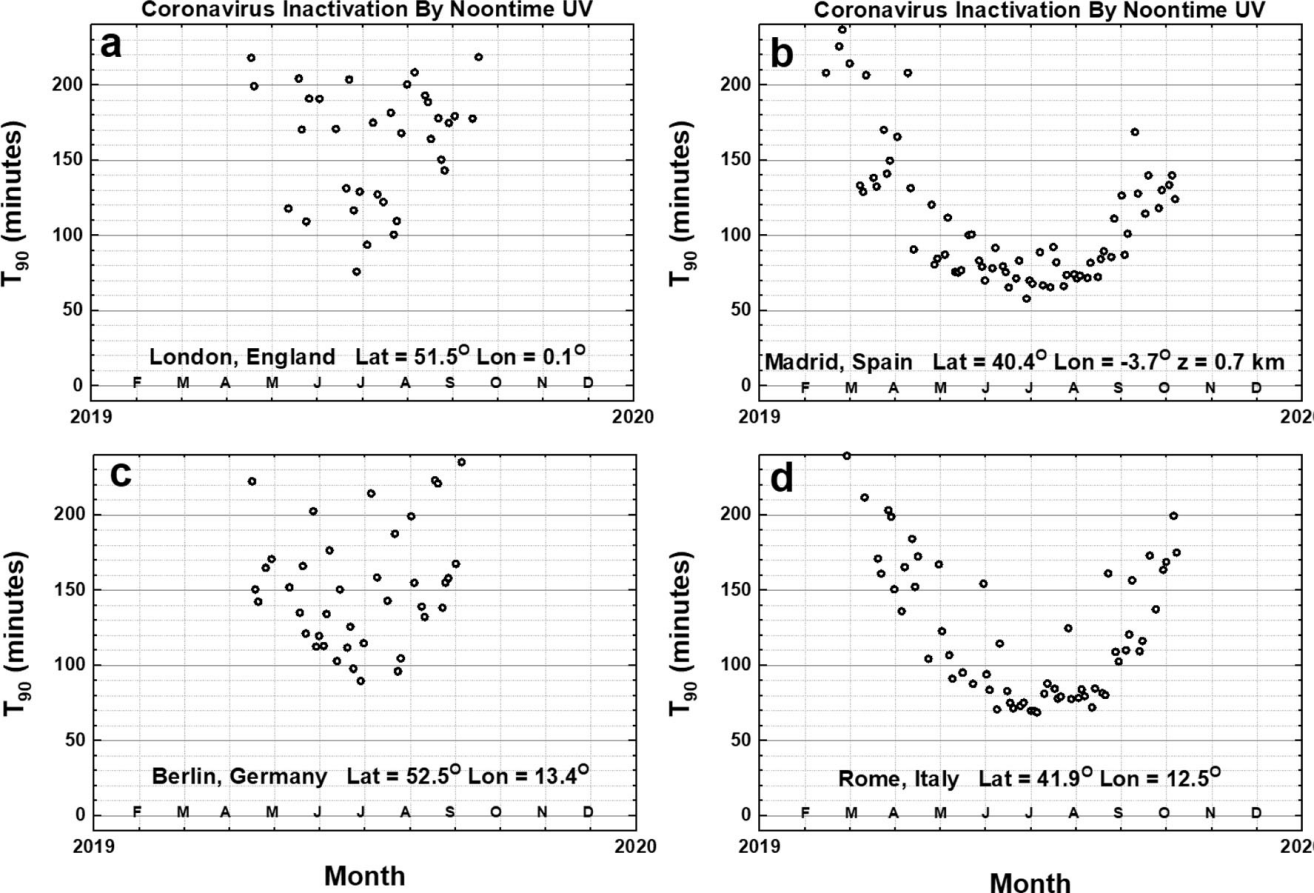
Noontime *T*_90_ coronavirus inactivation times for four Cities in Europe, London, UK; Madrid, ES; Berlin, DE; and Rome, IT, using *D*_90_ = 40 J/m^2^. For the calculated RS SARS CoV-2 *D*_90_ = 3.2 J/m^2^, *T*_90_ in June would about 8 min for London, 5 min for Madrid, 8 min for Berlin, and 5 min for Rome. Figures are truncated at 240 min

When the *D*_90_ = 40 J/m^2^ calculation is applied to four European cities (Fig. 6) (latitudes 40^O^ to 53^O^), the periods of 60–80 min inactivation are reduced to where London, England’s and Berlin, Germany’s shortest *T*_90_ inactivation times are close to 70–90 min and quickly increase as the local time differs from noon or the month differs from the June solstice (*θ* increasing). Two other cities, Rome, Italy and Madrid, Spain, have *T*_90_ values similar to New York City and have minimum *T*_90_ of 60 to 70 min in June, July, and August.

**Fig. 7:**
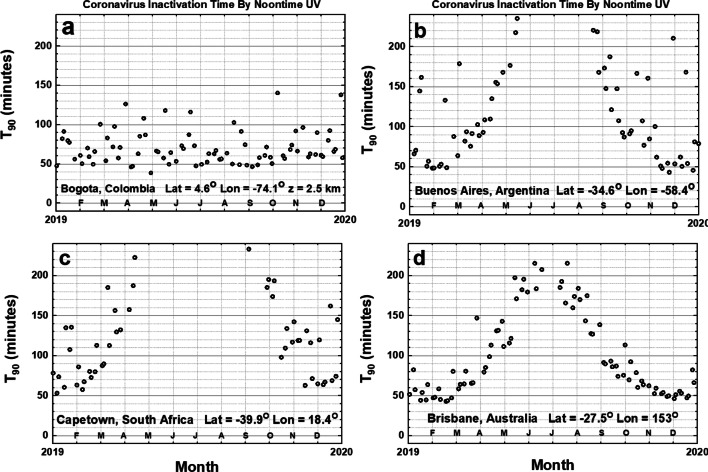
Noontime *T*_90_ for an equatorial region city, Bogota, CO, and three cities in the Southern Hemisphere: Buenos Aires, AR; Cape Town, ZA; and Brisbane, AU using *D*_90_ = 40 J/m^2^. For the calculated RS SARS CoV-2 *D*_90_ = 3.2 J/m^2^, *T*_90_ in June would about 4 min for Bogota, in January 4 min Buenos Aires, Capetown, and Brisbane. Figures are truncated at 240 min

Figure 7 shows the lack of significant *θ* dependence for a city in the equatorial zone; Bogota, Colombia at an altitude of 2.5 km with *T*_90_ = 50 min on many days of the year. The remaining three cities in Fig. 7 are in the Southern Hemisphere, which means their summer period is shifted 6 months with the minima of *T*_90_ occurring in December and January. *T*_90_ for Cape Town, South Africa at 39.3^O^ S behaves in a manner similar to New York City at 40.7^O^ N. For the sites shown in Figs. 6 and 7 minimum noontime *T*_90_ is less than 10 min for the SARS CoV-2 virus with *D*_90_ = 3.2 J/m^2^.

**Fig. 8:**
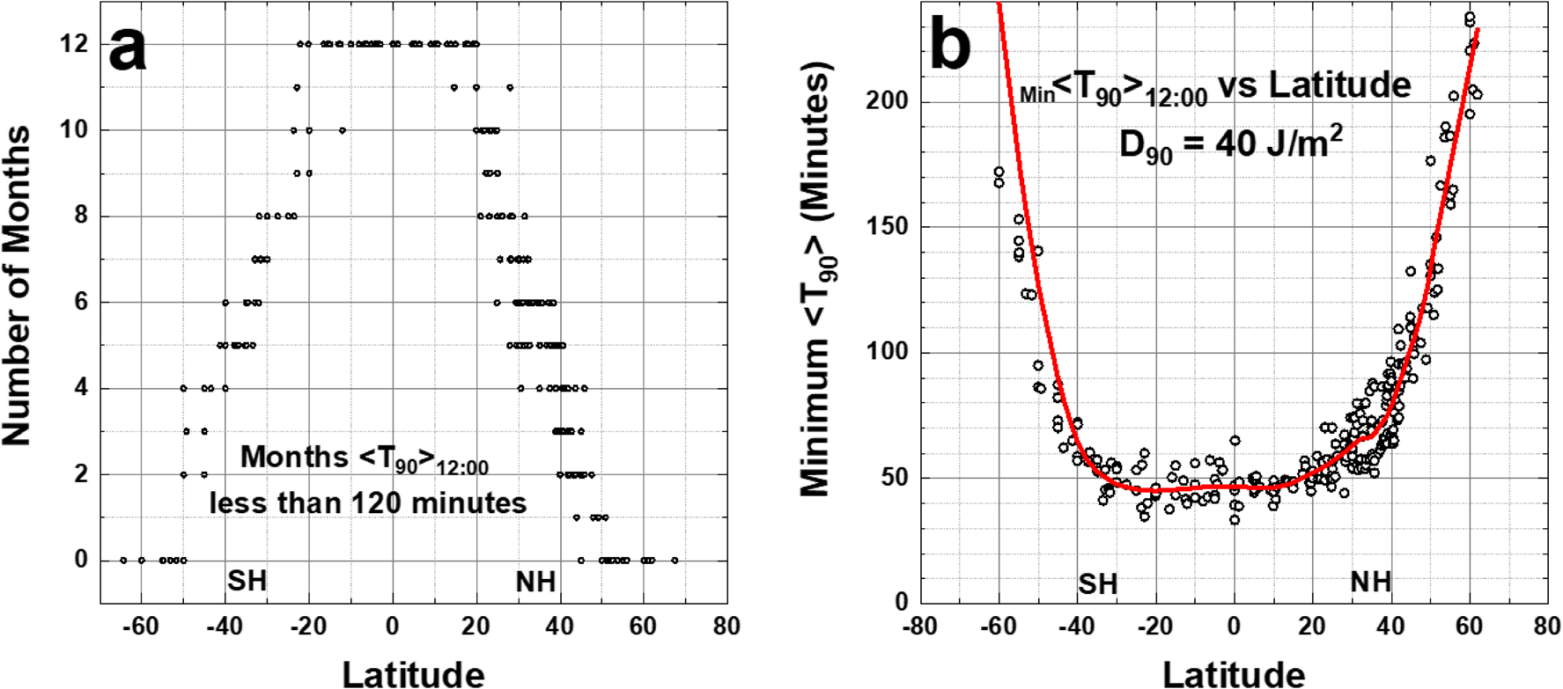
**a** Number of months <*T*_90_> _12:00_ ≤ 120 min from column 6 in Table s1. **b** Minimum _Min_ <*T*_90_> _12:00_ vs. latitude from the data from column 5 in Table s1 as a function of latitude. The smoothed curve is a Loess(0.3) fit to the data. Loess(f) is locally weighted least squares fit to a fraction f of the data points, (Cleveland 1979, 1981). Figure is truncated at 240 min

Figure 8 summarizes the 190 city table (Online Resource 3: Table S1). Almost all the equatorial zone cities show 12 months of <*T*_90_> _12:00_ ≤ 50 min. The number of months for <T_90_> _12:00_ ≤ 120 min decreases with increasing latitude. In Fig. 8a, the rate of decrease for the Northern Hemisphere (NH) is 3.0 months per 10^O^ and in the Southern Hemisphere (SH) it is 2.6 months per 10^O^ of latitude away from the equatorial zone, although there are fewer points to accurately determine the SH slope.

The equatorial zone shows that most of the minimum values of _Min_ <*T*_90_> _12:00_ are less than 50 min (Fig. 8b) up to about ± 25^O^ latitude for sites with fewer cloudy days per month. For latitudes outside the equatorial zone, where both the ozone amounts and SZA are larger, _Min_ <*T*_90_> _12:00_ increases. High mountain area sites also have lower noontime minimum _Min_ <*T*_90_> _12:00_ than sea-level sites.

## Estimates of variation with time of day for minimum <*T*_90_>

**Fig. 9:**
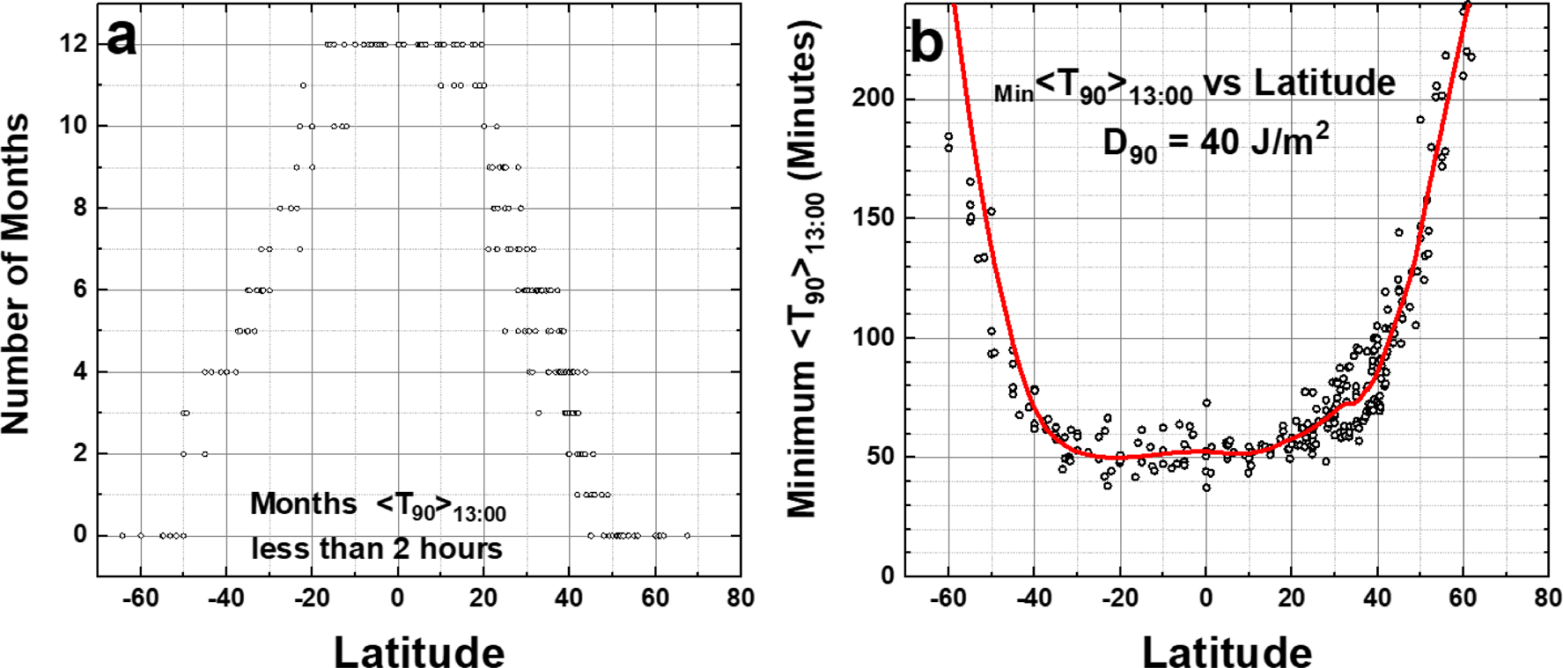
**a** Number of months <*T*_90_> _13:00_ ≤ 120 min. **b** 13:00 h minimum _Min_ <*T*_90_> _13:00_ vs. latitude. The smoothed curve is a Loess(0.3) fit to the data. Figure is truncated at 240 min

**Fig. 10:**
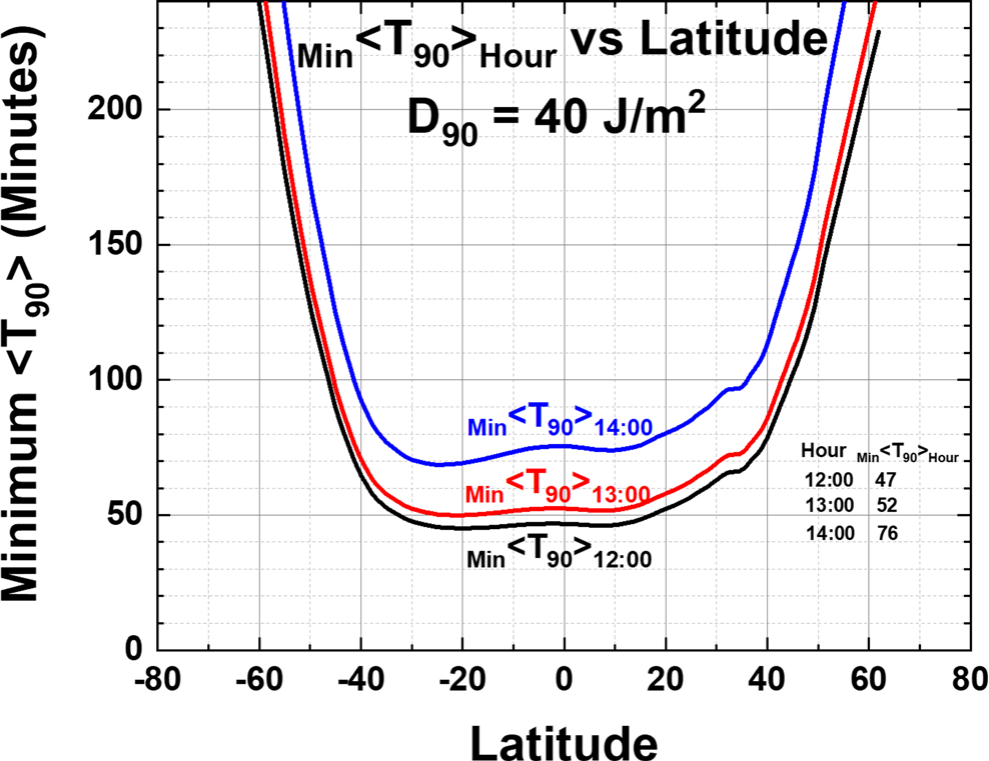
Comparison of Loess _Min_ <*T*_90_> _Hour_ for 12:00, 13:00, and 14:00 h. The values at *θ* = 0^O^ are _Min_ <*T*_90_> _Hour_ = 47, 52, 76 min at 12:00, 13:00, 14:00 h, respectively. Figure is truncated at 240 min

The increase of *T*_90_ with SZA is shown in Fig. 2 for noontime irradiances. The same effect applies to other times of the day when *θ* increases relative to noon (Figs. 9 and 10). For solar times away from noon, the minimum of monthly averages _Min_ <*T*_90_> increases such that _Min_ <*T*_90_> 14:00> 70 min for all mid-latitude cities before 10:00 and after 14:00 h solar time. However, at 11:00 and 13:00 h, there are still a significant number of mid-latitude sites with _Min_ <*T*_90_> _13:00_ < 75 min and equatorial sites where _Min_ <*T*_90_> _13:00_ < 60 min (Fig. 9a) for 2 or more months. Note that calculated _Min_ <*T*_90_> _10:00_ = _Min_ <*T*_90_> _14:00_ and _Min_ <*T*_90_> _11:00_ = _Min_ <*T*_90_> _13:00_.

Figure 10 compares the smoothed Loess (0.3) curves from Figs. 8b and 9b, plus a similar calculation for 14:00, showing the effect of time of day (12:00 to 14:00) on the minimum _Min_ <*T*_90_> _Hour_. The difference between _Min_ <*T*_90_> _13:00_ and _Min_ <*T*_90_> _12:00_ is from 5 to 10 min for sites between ± 30^O^ latitude extending to 25 to 30 min at 14:00.

**Fig. 11:**
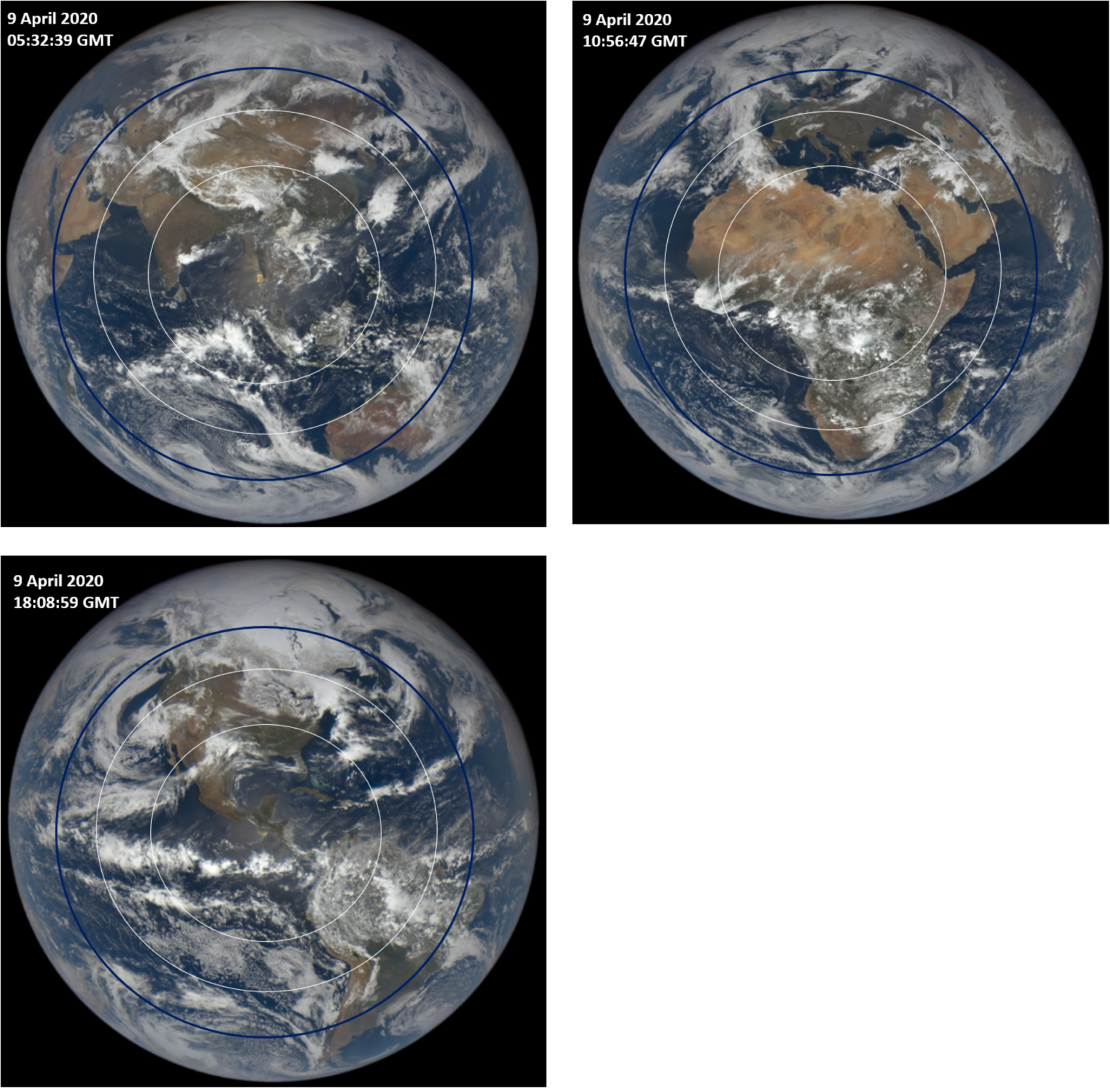
Earth images from DSCOVR/EPIC for April 9, 2020 showing the superimposed outer circle (white) of clear-sky _Min_ <*T*_90_> less than 65 min and the inner circle (white) for _Min_ <*T*_90_> less than 50 min when *D*_90_ = 40 J/m^2^. Sunrise is on the left (west) and sunset is on the right (east). The subsolar point is approximately in the center of the circle slightly offset by the small DSCOVR/EPIC orbital view angle. For the calculated RS SARS CoV-2 *D*_90_ = 3.2 J/m^2^, a near-circle (dark-line) with solar zenith angle < 60^O^ centered on the subsolar point would have _Min_ <*T*_90_> less than 20 min

Using the SARS CoV results from Figs. 2 and 10, it is possible to estimate the regions on the Earth where _Min_ <*T*_90_> _Hour_ < 50 min and _Min_ <*T*_90_> _Hour_ < 65 min and superimpose these criteria on color images from the DSCOVR/EPIC spectroradiometer to show the seasonal dependence driven by changes in the solar declination angle *δ*. Figure 11 shows solar illuminated Earth color images https://epic.gsfc.nasa.gov/ from sunrise to sunset obtained at the stated Greenwich Mean Time (GMT) for April 9, 2020 with the superimposed outer white circle representing the calculated 65-min _Min_ <*T*_90_> _Hour_ and the inner white circle the 50-min _Min_ <*T*_90_> _Hour_. Additional EPIC images are obtained approximately every 65 min (NH summer) to 108 min (NH winter) as the Earth rotates.

**Fig. 12:**
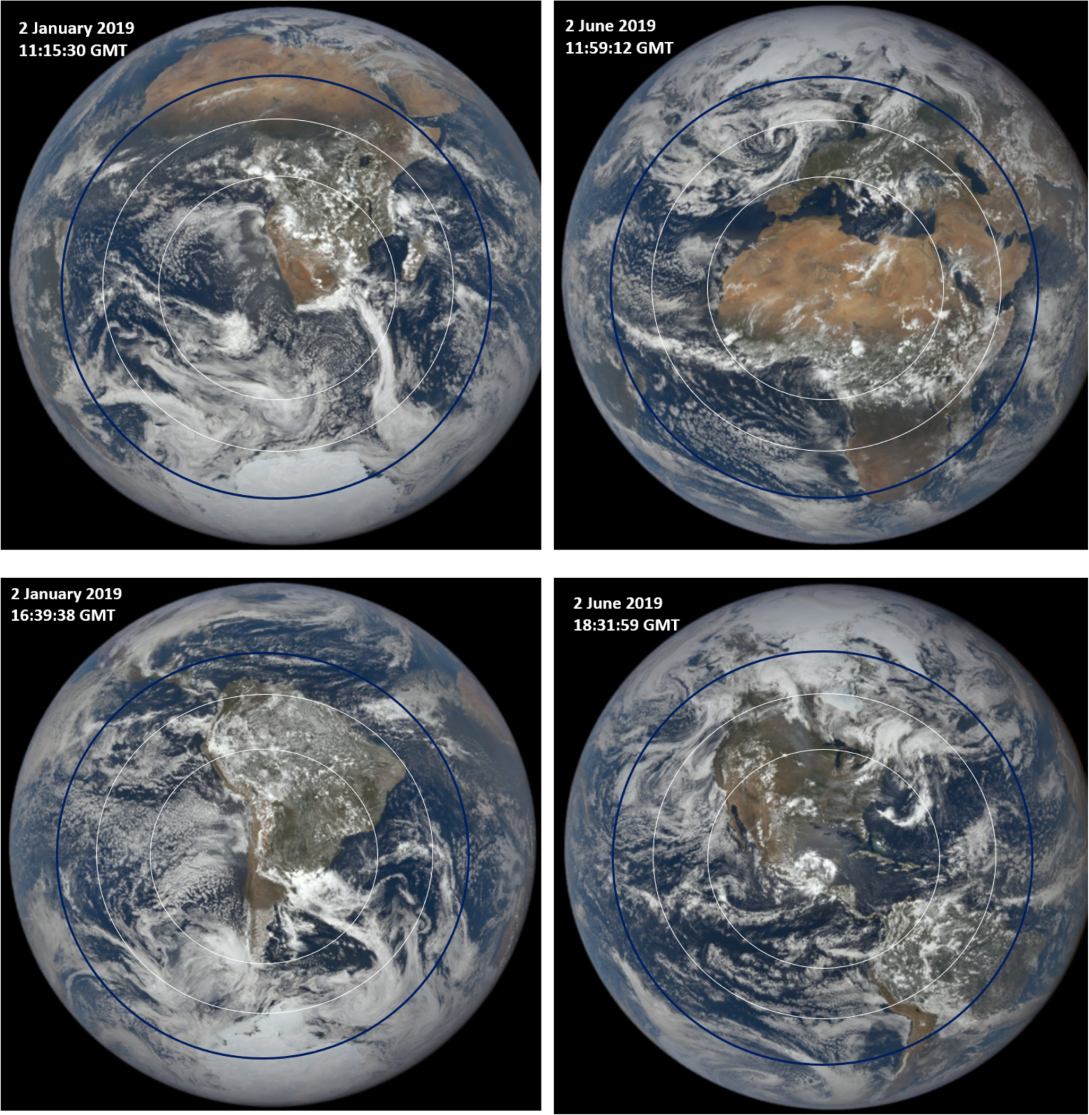
Earth images from DSCOVR/EPIC for January 2, 2019 and June 2, 2019 showing the superimposed outer circle (white) of clear-sky _Min_ <*T*_90_> less than 65 min and the inner circle (white) for _Min_ <*T*_90_> less than 50 min when *D*_90_ = 40 J/m^2^. Sunrise is on the left (west) and sunset is on the right (east). The subsolar point is approximately in the center of the circle slightly offset by the small DSCOVR/EPIC orbital view angle. For the calculated RS SARS CoV-2 *D*_90_ = 3.2 J/m^2^, a near-circle (dark-line) with solar zenith angle < 60^O^ centered on the subsolar point would have _Min_ <*T*_90_> less than 20 min

The images (Figs. 11 and 12) contain the subsolar point *θ* = 0^O^ with increasing *θ* radially in all directions away from the subsolar point. Since April 9 is 18 days after the March equinox, the subsolar point is about 7.6^O^ north of the equator. At 05:32:39 GMT, the clear-sky *T*_90_(65 min) circle covers all of China and extends into southern Russia, Japan, and Korea. At 10:56:47 GMT, the outer circle covers southern Europe. Approximately 7 h later at 18:08:59 GMT, as the Earth rotates 15^O^ longitude per hour, EPIC is viewing North and South America where the 65-min _Min_ <*T*_90_> _Hour_ circle extends as far north as the border of Canada (about 48^O^ N) and as far south as the northern border of Chile (about 18^O^ S).

Figure 12 shows Earth images from days near the solstices in January and June 2019. The image from January 2, 2019 at 16:38:38 GMT shows the 65-min _Min_ <*T*_90_> _Hour_ circle extending to the middle of Africa and well south of Cape Town, South Africa. In contrast, on June 2, 2019 at 11:59:12 GMT, the subsolar point is near 23^O^ N and the 65-min _Min_ <*T*_90_> _Hour_ circle extends as far north as England and Germany. As the Earth rotates to later GMT, the 65-min _Min_ <*T*_90_> _Hour_ circle would contain all of South America and later, on the next calendar day, Australia and New Zealand.

Since the EPIC images are synoptic, the longitudes are equivalent to local solar time of day (15^O^/hour). For *D*_90_ = 40 J/m^2^, the circles at their widest point are about 45^O^ or 3 h. This means that inactivation times are less than 65 min for Δ*t* = 09:00 to 15:00 h for latitudes of the subsolar point in the equatorial region, ± 23.45^O^. The longitudinal 65-min inactivation time-interval Δ*t* decreases with increasing latitudinal distance away from the subsolar latitude *δ*, Δ*t* ≈ 3cos(*θ* - *δ)* hours for |*θ* - *δ*| ≤ 90^Ο^, where *δ* = solar declination angle. For the calculated RS SARS CoV-2 *D*_90_ = 3.2 J/m^2^, longitudinal 20-min *T*_90_ inactivation time-interval Δt ≈ 4cos(*θ* - *δ*) hours for |*θ* - *δ*| ≤ 90^Ο^.

## Estimate of *T*_90_ for SARS-CoV-2 causing COVID-19

**Fig. 13:**
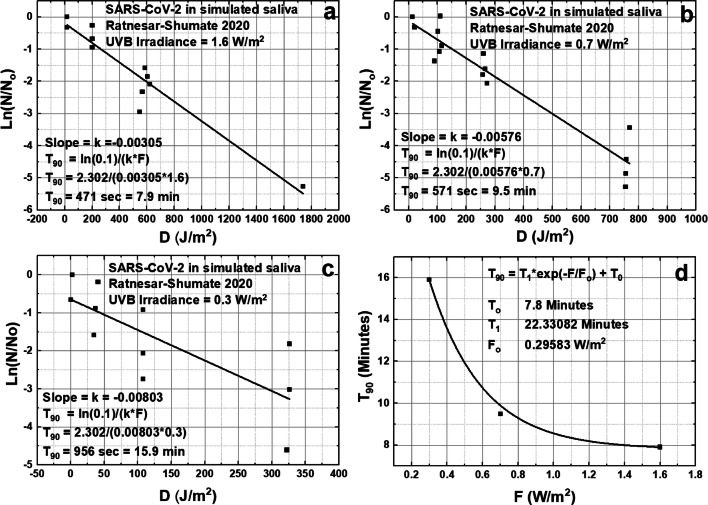
Inactivation of the SARS-CoV-2 virus at three different irradiances of simulated solar UVB, panels **a**, **b**, and **c**, 1.6, 0.7, and 0.3 W/m^2^ for the CoV-2 virus on a stainless-steel mesh surface based on the data from Fig. 4 of RS. Panel **d** is a summary of T_90_ from a, b, and c

**Fig. 14:**
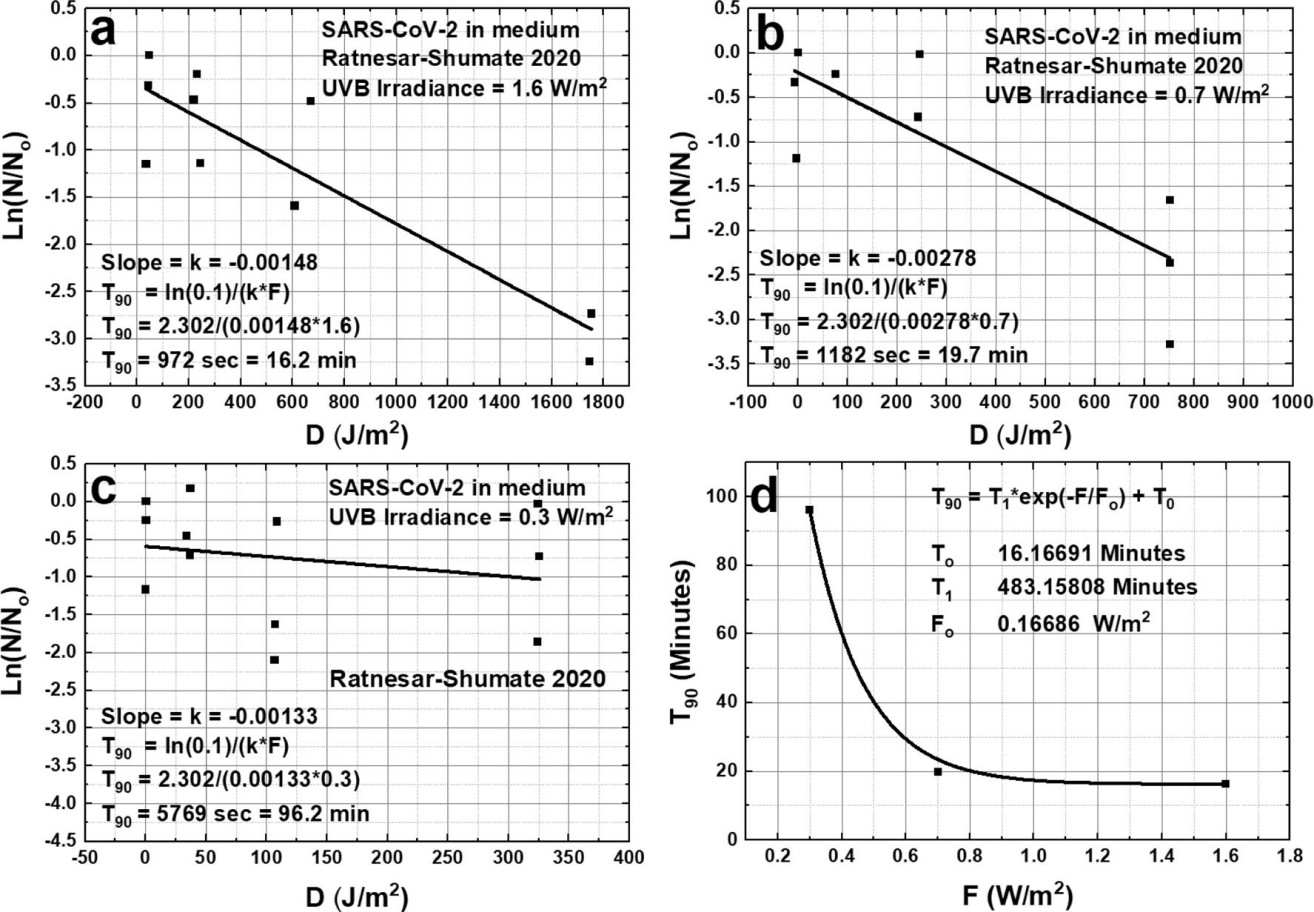
Inactivation of the SARS-CoV-2 virus at three different irradiances of simulated solar UVB, panels **a**, **b**, and **c**, 1.6, 0.7, and 0.3 W/m^2^ for CoV-2 virus suspended in a growth medium based on the data from Fig. 5 of RS. Panel **d** is a summary of T_90_ from a, b, and c

A recent study by Ratnesar-Shumate et al. (2020) performed laboratory studies of simulated June solstice solar UVB at 40^O^ N inactivation of the SARS-CoV-2 virus that causes the current COVID-19 pandemic. RS compared the simulated UVB to solar amounts at the Earth’s surface using the TUV radiative transfer model. Two basic experiments were run, one with droplets of virus in artificial saliva dried onto a stainless-steel mesh and the other using SARS-CoV-2 suspended in growth medium. As expected, it was found that inactivation times for virus suspended in a growth medium were significantly longer than for exposed virus on the steel mesh. Figures 13 and 14 show RS data (electronic digitization of RS Figs. 4 and 5) in natural logarithmic form, that is, in terms of *T*_90_ on the assumption that the exponential decay model (Eq. 3) is applicable.

6 $$ N/{N}_o=\exp \left(- kD\right)=\exp \left(- kPT\right) $$

7 $$ T=- Ln\left(N/{N}_o\right)/(kP)\kern0.5em {T}_{90}= Ln(0.1)/(kP) $$

In Eqs 6 and 7, P (W/m^2^) corresponds to Eq.1, *T* is the exposure time (seconds) and *D* is the UVB dose (J/m^2^), where *D* = PT. In RS, *P* is not weighted with an action spectrum. The slope k (Figs. 13 and 14) is determined from a least squares linear fit to the survival fraction Ln(N/N_o_) vs. the UVB dose *D* (J/m^2^) for three different exposure rates. When *P* is small, the determination of *k* from Ln(N/N_o_) is less certain.

In Fig. 13, the received UVB dose *D* is plotted against Ln(N/N_o_) for three different UVB irradiances. Of these, the slope *k* for *P* = 0.3 W/m^2^ has the largest uncertainty *k*(0.3) = − 0.00803 ± 0.00221 compared to *k*(1.6) = − 0.00305 ± 0.00031. The result is *T*_90_(1.6) = 7.9 min, *T*_90_(0.7) = 9.5 min and *T*_90_(0.3) = 15.9 min, all short inactivation times. Note that *T*_999_ values (99.9%) are four times *T*_90_, which are still less than 1 h. These *k* estimates are slightly different than those in RS (6.8, 8.0, and 12.8 min, respectively), but do not significantly affect the current analysis and conclusions. The differences probably arise from different weighting of data points when RS’s exposure times are almost zero (see RS’s Figs. 4 and 5).

If it is assumed that the action spectrum *A*(*λ*) applies, then the 254 nm UVC *D*_90_ equivalent is approximately DE_90_ = 3.2 J/m^2^ (Table 1) when using TUV calculations to approximate their result for 40^O^ N on June 21 at noon. Using the DE_90_ value permits easy estimates of *T*_90_ for a wide range of geographic and atmospheric conditions. There are some differences between the simulated RS solar spectrum and the spectrum calculated here from TUV for different SZA in this study. The main difference is that the peak sensitivity shifts towards longer wavelengths (Fig. 2) as SZA or TCO_3_ increases. Estimates of *T*_90_ for SARS CoV-2 are not significantly affected by the choice of *A*(*λ*), since the value of *D*_90_ was adjusted to match the simulated UVB amounts in RS. RS measurements made in simulated sunlight give an error estimate of ± 10% (their Fig. 5). Estimating DE_90_ requires combining the two independent errors giving an error of 22%, or DE_90_ = 3.2 ± 0.7 J/m^2^.

When the same exposures are applied to the virus in a growth medium the RS-based results are shown in Fig. 14. *T*_90_(1.6) = 16.2 min and *T*_90_(0.7) = 19.7 min compared to RS’s values of 14.3 and 17.6. As above, using the action spectrum *A*(*λ*) gives a 254 nm UVC *D*_90_ equivalent of approximately 6.5 ± 1.4 J/m^2^ (Table 1).

A recent analysis for the SARS CoV-2 inactivation times (Sagripanti and Lytle 2020) obtains an estimate for *D*_37_ = 3.0 J/m2, which translates to *D*_90_ = 3 ln(0.1)/ln(0.37) = 6.9 J/m^2^, larger than the estimate for *D*_90_ = 3.2 J/m^2^ given above. The methods for obtaining *D*_90_ are entirely different. The method used here relies on finding a value of 254 nm *D*_90_ that yields approximately the same inactivation time, 6.8 min, as RS finds for midday during the summer solstice at 40^O^ N latitude using the same TUV radiative transfer code. Sagripanti and Lytle (2020) infer their value from laboratory measurements of viruses with a similar genomic structure and “the fact that UVC sensitivities of viruses depends proportionally on genome size, especially with single-stranded RNA or DNA.” Most of the measurements they reference were made with viruses in a liquid medium and should be compared to the value obtained from RS data, using the radiative transfer method, of 6.5 J/m^2^ for viruses in a growth medium.

The values of *T*_90_ estimated by Sagripanti and Lytle (2020) are 3 to 4 times larger than estimated here. Part of the difference arises from their estimate of *D*_90_ being 2.15 times larger. The remainder must come from the estimate of noontime solar flux entering into ʃ*F*(*λ*) *A*(λ)*d λ* in Eq. 1. They use an approximation to the noontime solar flux based on 35% of the daily fluence occurring during a 2-h period surrounding solar noon. “Thus, 35% of the total daily UVB fluence divided by 120-min yields the noontime UVB flux(in J m^−2^ min^−1^).” The noontime F(λ) in Eq 1 is calculated using the SZA and local atmospheric parameters for each site estimated from OMI data, which may differ from the 35% estimate.

**Fig. 15:**
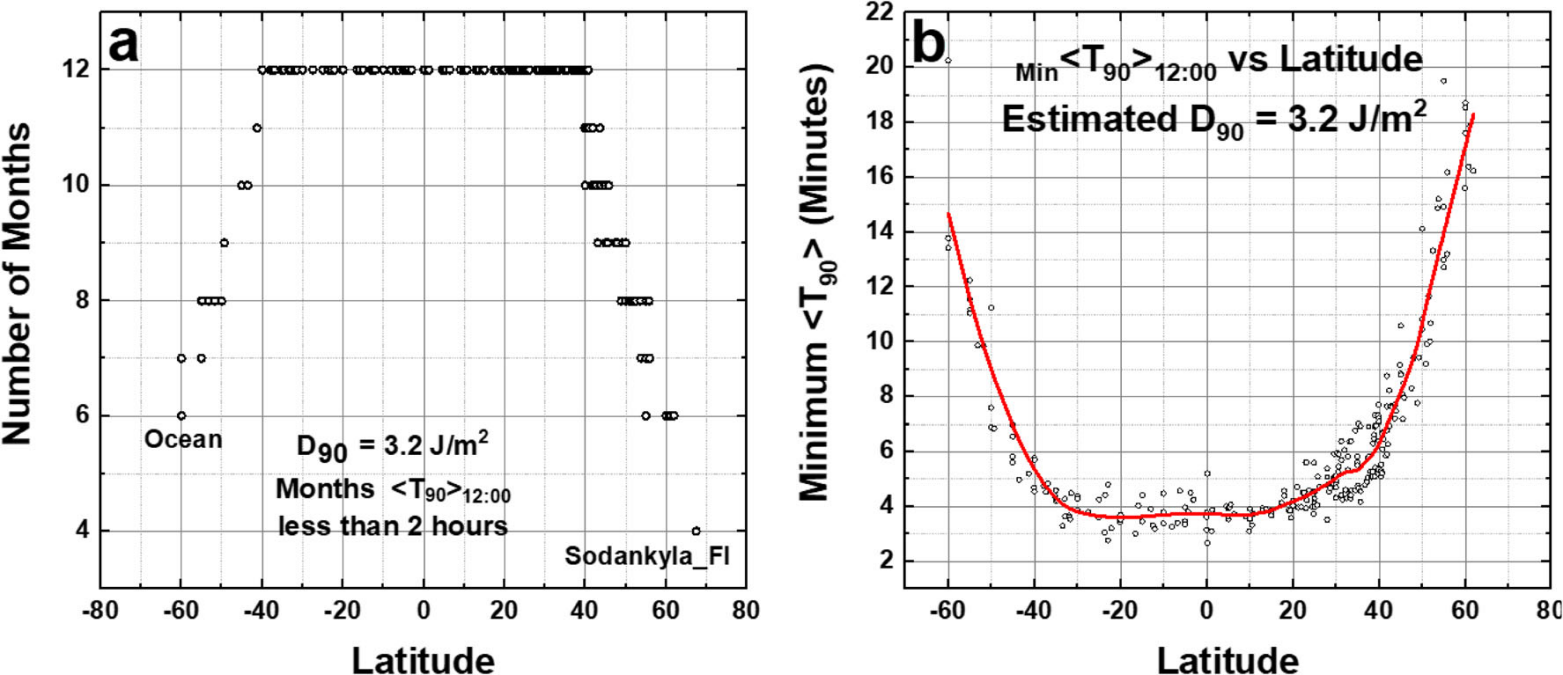
**a** Number of months _Min_ <*T*_90_> _12:00_ ≤ 120 min (column 8 Table s1). **b** Minimum _Min_ <*T*_90_> _12:00_ vs. latitude as a function of latitude (column 7 Table s1). The smoothed curve is a Loess(0.3) fit to the data. For the estimated RS SARS CoV-2 *D*_90_ = 3.2 J/m^2^, *T*_90_ values at all latitudes − 60^O^ to 60^O^ are less than 18 min and the minimum values at the equator are *T*_90_ = 4 min

If RS’s smaller values for *T*_90_ are used instead of the values in Figs. 13 and 14, then the UVC equivalent would be smaller than *D*_90_ = 3.2 J/m^2^ estimated here. The value *D*_90_ = 3.2 J/m^2^ is approximately 12.5 times smaller than the UVC *D*_90_ = 40 J/m^2^ for the SARS CoV virus used in the previous sections leading to *T*_90_ of about 4 min at the equator and about 5 min at 40^O^ N during the summer solstice (Fig. 15b). The main conclusion that SARS CoV-2 virus is quickly inactivated by UVB in sunlight remains unchanged. For estimating day-to-day inactivation times, the exact *T*_90_ numbers for SARS CoV-2 virus are unimportant on any given day because of the larger *T*_90_ variability caused by significant atmospheric transmission changes even on days that appear relatively clear of clouds and aerosols.

The RS measurements show that *T*_90_ for SARS CoV-2 on a surface is smaller than in a growth medium, which is similar to the results for the airborne MHV virus *D*_90_ = 6.6 J/m^2^ and the same virus in liquid, *D*_90_ = 95 J/m^2^, with the value in liquid (Table 1) much greater than the value in air.

**Fig. 16:**
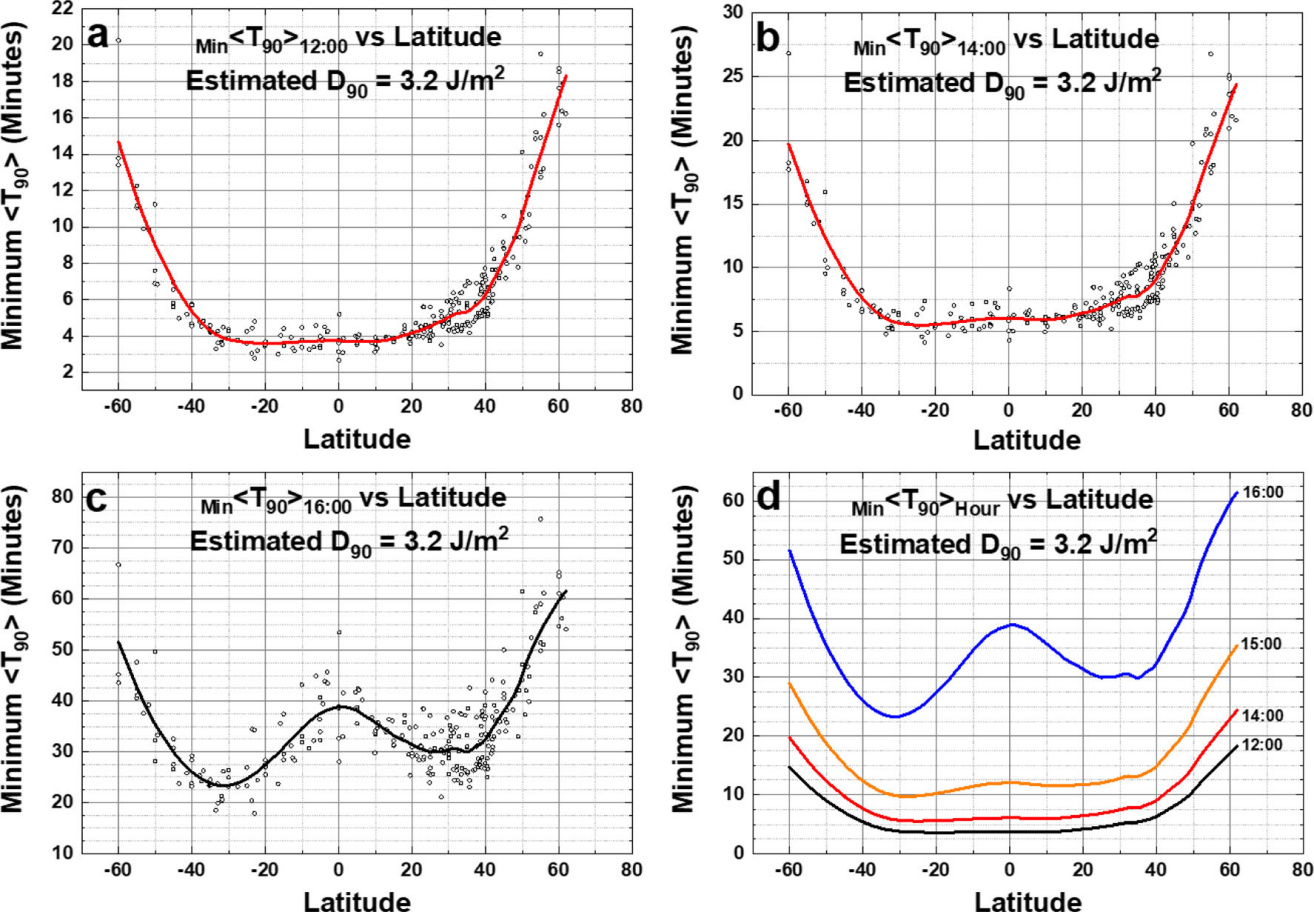
Estimated inactivation times for SARS Cov-2 virus dried on surfaces as a function of latitude at 12:00, 14:00, and 16:00 local solar time for the calculated RS *D*_90_ = 3.2 J/m^2^. **a** 12:00, **b** 14:00, **c** 16:00, **d** compare four different hours

Figure 16 shows the inactivation times for SARS CoV-2 virus on surfaces by solar UVB for three different times of the day when *D*_90_ = 3.2 J/m^2^, which is the approximate equivalent RS’s laboratory simulated solar UVB. The results show minimum inactivation times _Min_ <*T*_90_> increasing as |LST–12:00| increases, but always less than 1 h. For high latitude sites considered (latitude ≥ 60^O^) during the winter months *T*_90_ inactivation times are much longer than 2 h and, on many days, no inactivation is possible. For example, Sodankylä, Finland has only 4 months during which the inactivation time *T*_90_ < 2 h (Fig. 15a).

As the time of day increases to 16:00 LST a peculiar SZA effect occurs because of the spherical geometry. The minimum SZA for near solstice conditions shifts to higher latitudes near 40^O^ north and south, causing _Min_ <*T*_90_> _14:00_ to be smaller at 35^O^ S and 35^O^ N than it is near the equator. Note that these are annual minimum *T*_90_ that includes both summer solstices in their respective hemispheres giving rise to two minima.

## Summary and discussion

A study of coronavirus inactivation times by UV solar irradiation is presented for two classes of experimental laboratory data. First, are those measurements made at 254 nm and extrapolated to wavelength longer than 290 nm using an action spectrum *A*(*λ*) (Lytle and Sagripanti 2005), and second, are the measurements made in simulated sunlight (Ratnesar-Shumate et al. 2020) that do not require the use of an action spectrum. For the RS case, *A*(*λ*) is used to estimate the value of 254 nm *D*_90_ that gives approximately the same *T*_90_ derived by RS for a June solstice at 40^o^ N (254 nm *D*_90_ = 3.2 ± 0.7 J/m^2^ for dried virus droplets on a steel mesh surface and 6.5 ± 1.4 J/m^2^ for viruses in a growth medium).

A fast calculation method, which closely approximates TUV radiative transfer results for clear and cloudy scenes, has been used for calculating 90% inactivation times *T*_90_ for SARS CoV and SARS CoV-2 viruses in a realistic atmosphere when exposed to sunlight based on 90% inactivation doses *D*_90_ at 254 nm. The method uses OMI satellite data for cloud transmission, ozone, and aerosol absorption over a wide range of latitudes, longitudes, and day of the year. For SARS CoV, a nominal value *D*_90_ = 40 J/m^2^ is used for 90% inactivation at 254 nm combined with the assumed applicable virus inactivation action spectrum *A*(*λ*) provided by Lytle and Sagripanti (2005). The results are used to calculate midday amounts of UVB from sunlight that can deactivate coronaviruses on horizontal surfaces by 90% in moderate amounts of time, *T*_90_ < 90 min at mid-latitudes, for low latitudes *T*_90_ < 60 min and for equatorial region sites *T*_90_ < 50 min. The SARS CoV *D*_90_ = 40 J/m^2^ model suggests that outdoor horizontal surfaces that have been unoccupied for at least 90 min and exposed to clear-sky midday levels of UVB sunlight are likely to have coronaviruses 90% inactivated during the Spring through Autumn months for mid- and low-latitude sites where *T*_90_ ≤ 90 min, and all year for equatorial sites. *T*_90_ results are also presented for different times of the day over a wide range of latitudes and SZA. Estimates are given for the number of months in each year that a given location has *T*_90_ < 2 h (Figs. 9, 10, 15, 16, and Table s1).

Inactivation by sunlight to undetectable levels of virus will take much longer, perhaps 2 to 3 times longer based on laboratory inactivation by UVC. For aerosolized particles carrying the SARS CoV virus, *D*_90_ = 7.11 J/m^2^ (Walker and Ko (2007), the value for the *T*_90_ value is reduced by a factor of 40/7.11 = 5.63. Of course, viruses deposited late in the day may persist overnight with inactivation delayed until the following day. The presence of common light to moderate cloud cover, LER < 0.3, increases the inactivation time as shown by the scatter in Figs. 5, 6, and 7.

For the recent Ratnesar-Shumate et al. (2020) laboratory results, the calculated equivalent 254 nm SARS CoV-2 *D*_90_ = 3.2 ± 0.7 J/m^2^. Calculated *T*_90_ < 7 min at mid-latitudes, while for the equatorial region sites *T*_90_ < 4 min, the reduction in *T*_90_ is a factor of 12.5 compared to using *D*_90_ = 40 J/m^2^. For the calculated RS SARS CoV-2 *D*_90_ = 3.2 J/m^2^, minimum inactivation times are less than 20 min for local solar times from 10:00 to 14:00 h and less than 60 min solar zenith angels *θ* < 60^O^ from the subsolar latitude for 08:00 to 16:00 h. For those surfaces that are near direct sunlight, but not in direct sunlight, there is ample diffuse UVB sunlight to inactivate SARS CoV-2 coronaviruses with about 70% more exposure, or less than 2 h for mid-latitudes and for equatorial sites in less than 90 min. At other times of the day between 11:00 and 13:00 local solar time, there are still many mid- and low-latitude sites with sufficient sunlight so that _Min_ <*T*_90_> _13:00_ ≤ 90 min. By 14:00 h, there are very few sites with _Min_ <*T*_90_> _14:00_ ≤ 90 min. During the summer solstices the _Min_ <*T*_90_> _12:00_ ≤ 65 min circles cover mid-latitude cities in both hemispheres. Cities at high latitudes greater than 60^O^ do not have periods where the inactivation times are less than 2 h. Unoccupied midday surfaces will become relatively virus free in short periods from Spring to Autumn. While sunlight will inactivate the SARS CoV-2 virus responsible for COVID-19, the midday UVB 90% inactivation time, 7 to 20 min, is too slow to protect against transmission between people outdoors in crowds.

## Electronic supplementary materials

ESM 1 (DOCX 301 kb)

ESM 2 (DOCX 88 kb)

ESM 3 (DOCX 30 kb)

## Data Availability

All data used in this study are available in stated public archives, listed references, or included explicitly in the study.
