# Supplementary material for: Inactivation times from 290 to 315 nm UVB in sunlight for SARS coronaviruses CoV and CoV-2 using OMI satellite data for the sunlit Earth
Source: Air Qual Atmos Health. 2020 Sep 15;14(2):217–33. doi: 10.1007/s11869-020-00927-2 (PMC7490326; doi:10.1007/s11869-020-00927-2)
Supplement: Supplementary file 1 — (DOCX 301 kb) [file 11869_2020_927_MOESM1_ESM.docx]

Online Resource 1

**Estimation of D_90_ from laboratory measurements as shown in Table 1**

D_90_ is calculated assuming a log linear relationship between UVC exposure and inactivation (Eq. 3 and S1), with a slope k (m^2^/J).

D_90_ = Ln(0.1)/k = -2.302/k (J/m^2^) (S1)

| 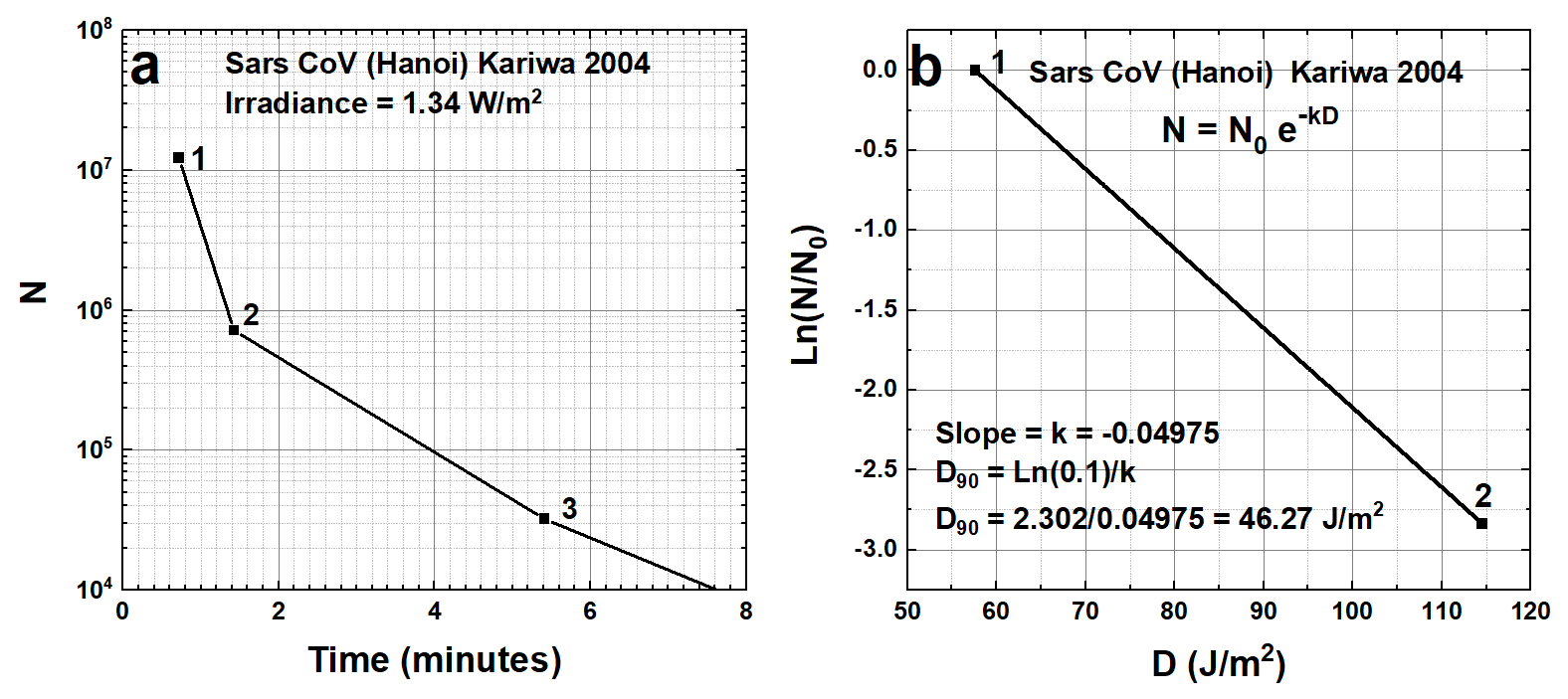 |
| --- |
| Fig. S1 Estimation of D_90_ from UVC inactivation of SARS CoV (Hanoi) Kariwa et al. (2004). a: The data shown in Kariwa et al. (2004). b: Determining the slope k between points 1 and 2. |

| 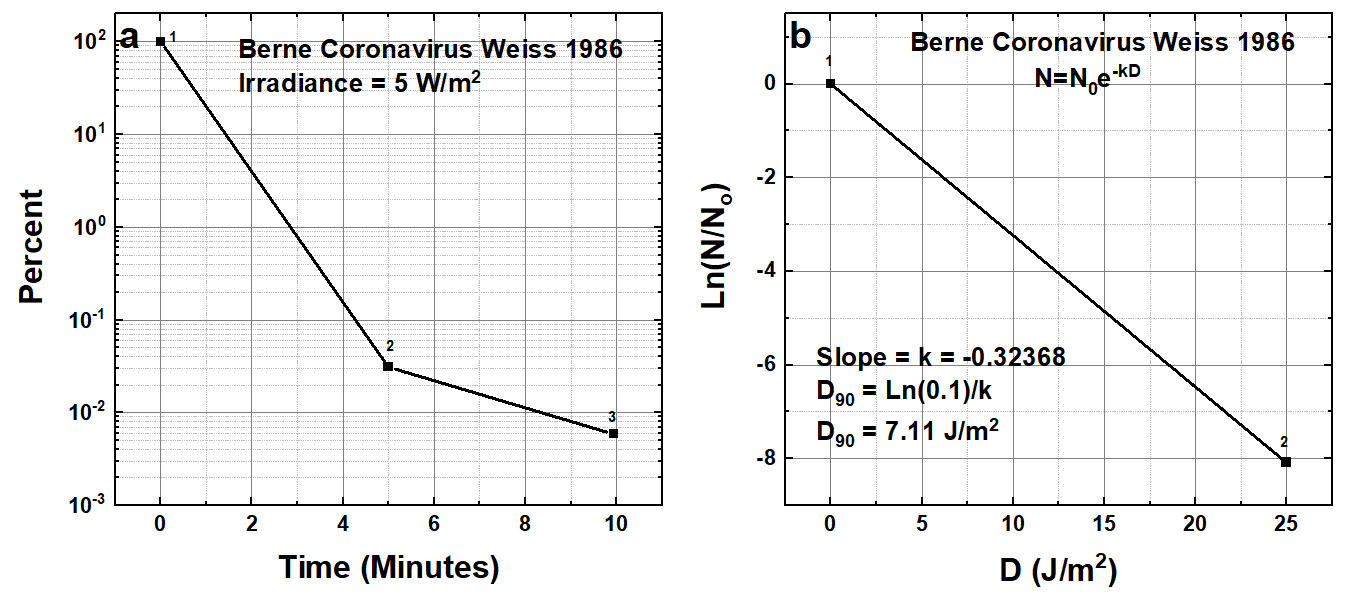 |
| --- |
| Fig. S2 Estimation of D_90_ from UVC inactivation of Berne-CV (Weiss and Horzinek 1986). a: The data shown in Weiss and Horzinek (1986). a: Determining the slope k between points 1 and 2. |

| 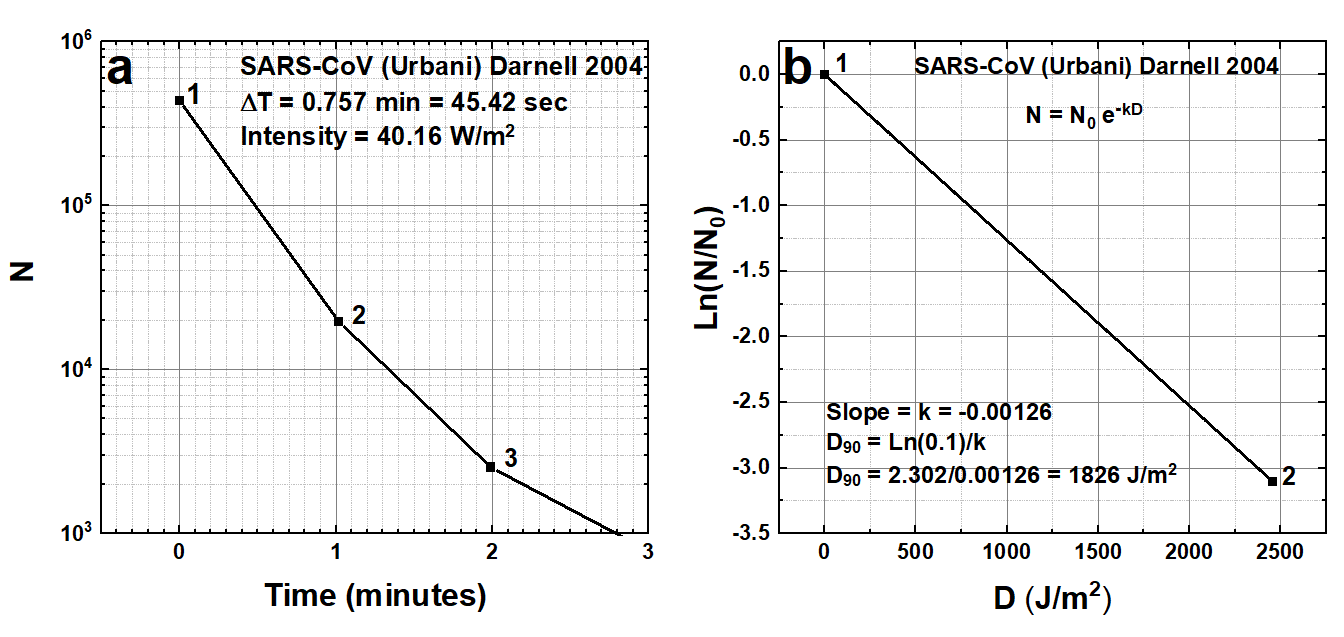 |
| --- |
| Fig. S3 Estimation of D_90_ from UVC inactivation of SARS-CoV (Urbani). a: Using the data shown in Darnell et al. (2004) b: Determining the slope k between points 1 and 2. |

| 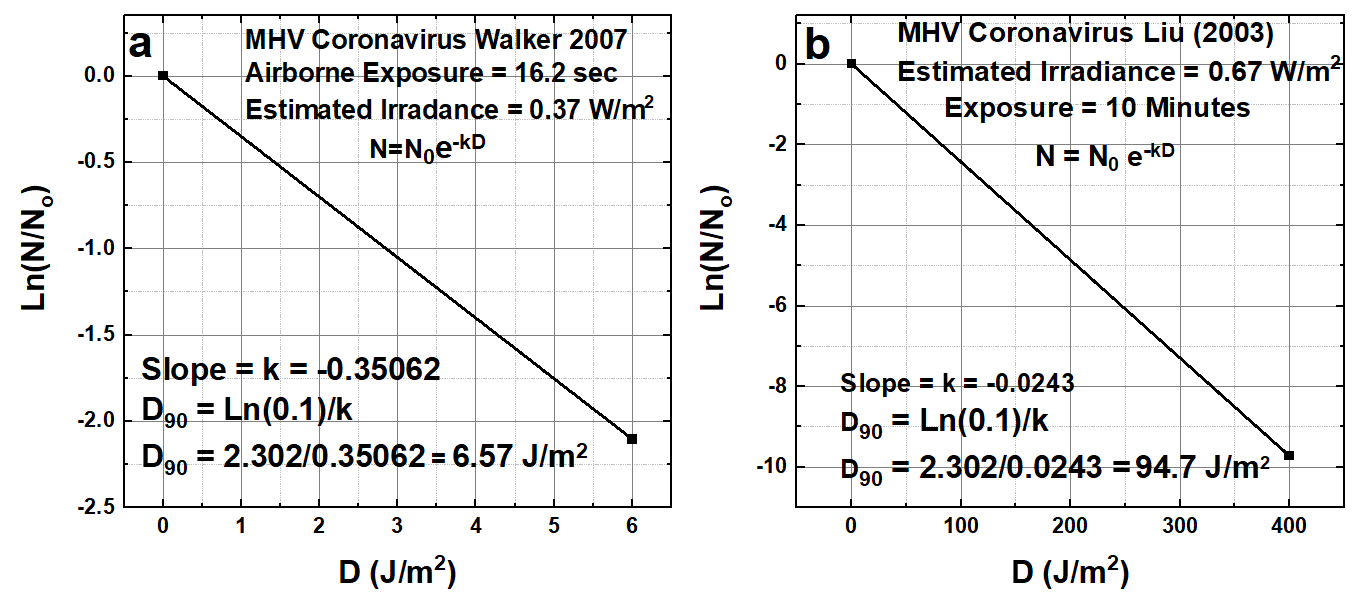 |
| --- |
| Fig. S4 Estimation of D_90_ from UVC inactivation of Murine Hepatitis Coronavirus (MHV). a: Airborne aerosol containing virus with a 16.2 second exposure to UVC at 0.37 W/m^2^ or a dose of 5.99 J/m^2^ (Walker and Ko (2007). b: MHV coronavirus inactivation under UVC exposure at 400 J/m^2^ in 1 mL liquid going from 100% to an undetectable level assumed to be 0.006% in 10 minutes (Liu et al., 2003). |

The estimated difference between the two MHV Coronavirus experiments suggest that the airborne virus may be much more susceptible to UVC inactivation than the same virus in a liquid environment. The value D_90_ = 94.7 J/m^2^ is uncertain, since Liu et al. (2003) do not state their minimum level of detectability after 10 minutes exposure. Kowalski et al. (2020) estimate D_90_ = 103 J/m^2^.
