# Supplementary material for: Inactivation times from 290 to 315 nm UVB in sunlight for SARS coronaviruses CoV and CoV-2 using OMI satellite data for the sunlit Earth
Source: Air Qual Atmos Health. 2020 Sep 15;14(2):217–33. doi: 10.1007/s11869-020-00927-2 (PMC7490326; doi:10.1007/s11869-020-00927-2)
Supplement: Supplementary file 2 — (DOCX 88 kb) [file 11869_2020_927_MOESM2_ESM.docx]

Online Resource 2

**Fast Calculation**

| $U(\theta)=\frac{a_{u}+c_{u}\theta^{2}+e_{u}\theta^{4}}{1+b_{u}\theta^{2}+d_{u}\theta^{4}}$  a_U_ = 0.02626230830607135  b_U_ = 0.0001769078685484589  c_U_ = -7.836485608356546X10^-06^  d_U_ = 3.779937799430998X10^-08^  e_U_ = 5.942420204940293X10^-10^ | (S2) |
| --- | --- |
| $R(\theta)=\frac{a_{r}+c_{r}\theta+e_{r}\theta^{2}}{1+b_{r}\theta+d_{r}\theta^{2}}$  a_r_ = 1.668045114523178  b_r_ = -0.01415788898583324  c_r_ = -0.02291337307048475  d_r_ = 6.362420107891145x10^-05^  e_r_ = 0.0001296005122963591 | (S3) |

The coefficients U(θ) and R(θ) for P_O_(θ,Ω) are expanded in rational fractions (Eq. S2,S3) with the fit such that the coefficient of determination r^2^ > 0.999. Note that the improved radiation amplification factor R(θ) is independent of the ozone value Ω. For θ = 0, the RAF is 1.668, which means a 1% increase in Ω will cause a 1.668% decrease in P_O_(0,Ω). Unlike the erythemal case (290-400nm), RAF_290–320_(θ) increases with increasing θ (Fig. S5).

The expressions for height dependence, cloud, and aerosol transmission are given in Eqs. S4-S6.

| H(z,θ) = 1+ (0.04937+1.67422x10^-4^θ -2.95768x10^-6^θ^2^+7.88211x10^-8^θ^3^) z 0 ≤ z ≤ 8 km | (S4) |
| --- | --- |
| C_T_ = (1 – LER)/(1 – R_G_) 0≤ LER ≤ 1 (Herman, 2010) | (S5) |
| C_A_ = F(t_A_ (l)) /F(t_A_=0) = 1/(1 + 3t_A_ (l)) (Herman et al., 2020) | (S6) |

R_G_ is the Total Ozone Mapping Spectrometer UV LER reflectivity of the Earth’s surface (Herman and Celarier, 1997) for locations without snow and ice. A global average value of R_G_ = 0.05. Equation S5 accounts for multiple reflections between a cloud and ground (Herman et al., 2009).

Aerosol fractional transmission C_A_ for UV irradiance is based on derived 354 nm aerosol optical depths and single scattering albedo from OMI measured radiances (Torres et al., 2007) both as a function of latitude and longitude. t_A_(305nm), near the peak of AF_O_ (Fig. 2), is based on the measured absorbing OMI aerosol optical depths t_A_(354nm) and absorption Angstrom exponent 1.8 (Eq. S7) derived from data obtained over Seoul, South Korea similarly to that derived for Santa Cruz, Bolivia (Mok et al., 2018).

|   (Herman et al, 2020) | (S7) |
| --- | --- |

Numerically, t_A_(305 nm) = 1.308 t_A_(354 nm).

SZA θ is determined from the standard spherical geometry equation (Eq. S8) based on latitude ζ, solar declination angle δ and local solar time t, -90^O^ ≤ ζ ≤ 90^O^, −23.45^O^ ≤ δ ≤ 23.45^O^, and 0 ≤ t ≤ 24 hours.

|  | (S8) |
| --- | --- |

At noon (local solar time = 12), cos(θ) = cos(ζ - δ) or θ = ζ - δ.

The solar declination angle δ changes with day of the year 1 ≤ D_OY_ ≤ 365, or 366 for leap years, and can be approximated by (Eq. S9)

| $\delta=-23.45^{o}\cos\left(360^{o}\frac{D_{OY}+10}{365}\right)$ | (S9) |
| --- | --- |

Since F_O_(λ) is computed for a solar distance of 1 AU, there is an approximate proportionality correction to the calculated solar irradiance at the top of the atmosphere D_S_ = 1/R_AU_^2^ for the distance from the sun relative to 1 astronomical unit AU versus day of the year D_OY_ (Eq. S10),

|  | (S10) |
| --- | --- |

The coefficient 0.0167 approximates the effect of ellipsoidal eccentricity of the Earth’s orbit. Equations 13 and 14 are standard approximations that are sufficiently accurate for this study.

| 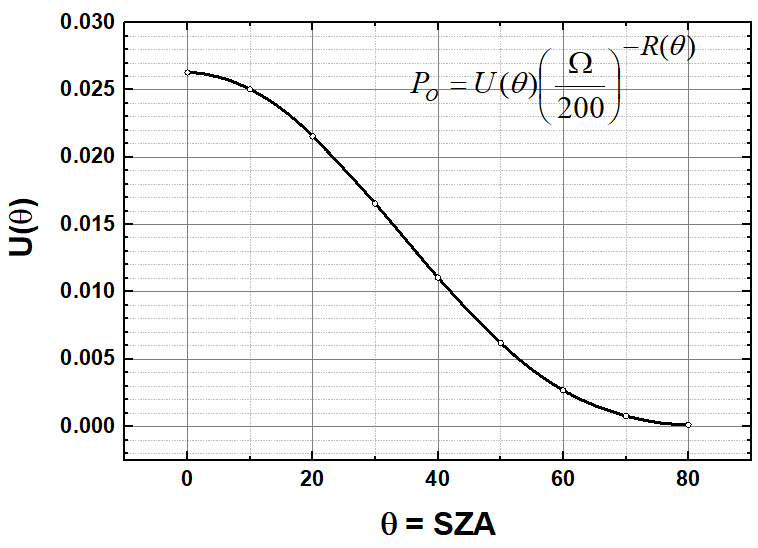 | 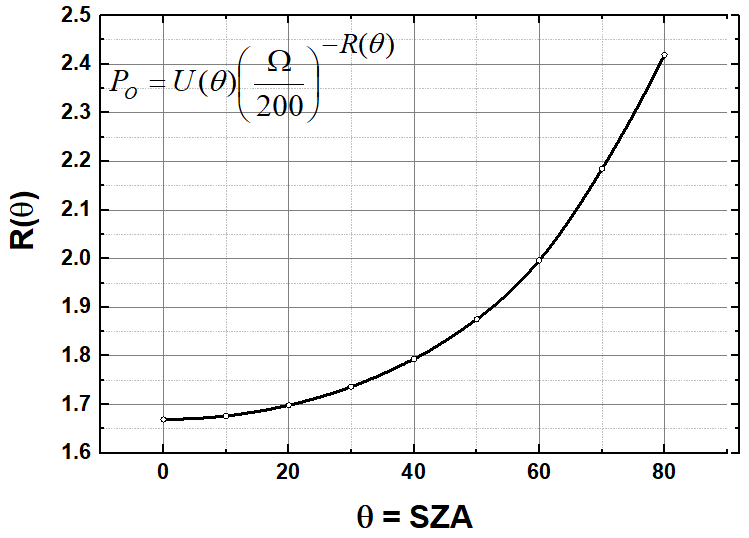 |
| --- | --- |
| Fig. S5A The fitting function U(θ) in Eq.5 | Fig. S5B The fitting function R(θ) in Eq.5 |

**Figure S5**

| 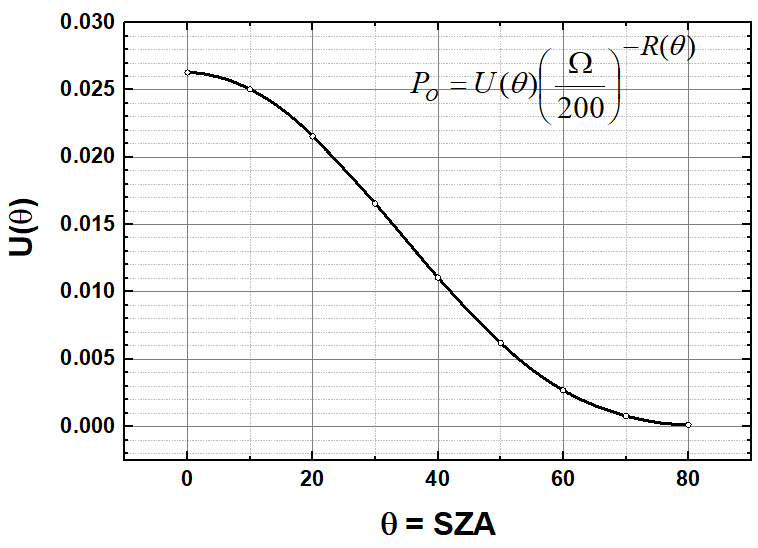 | $U(\theta)=\frac{a_{u}+c_{u}\theta^{2}+e_{u}\theta^{4}}{1+b_{u}\theta^{2}+d_{u}\theta^{4}}$  a_U_ = 0.02626230830607135  b_U_ = 0.0001769078685484589  c_U_ = -7.836485608356546X10^-06^  d_U_ = 3.779937799430998X10^-08^  e_U_ = 5.942420204940293X10^-10^ | (S1) |
| --- | --- | --- |
| Fig. S1A The fitting function U(θ) in Eq.5 |  |  |
| 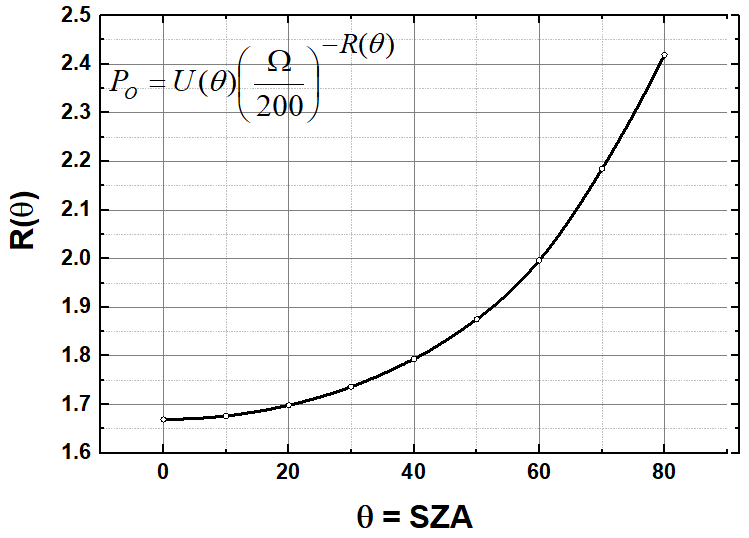 | $R(\theta)=\frac{a_{r}+c_{r}\theta+e_{r}\theta^{2}}{1+b_{r}\theta+d_{r}\theta^{2}}$  a_r_ = 1.668045114523178  b_r_ = -0.01415788898583324  c_r_ = -0.02291337307048475  d_r_ = 6.362420107891145x10^-05^  e_r_ = 0.0001296005122963591 | (S2) |
| Fig. S1B The fitting function R(θ) in Eq.5 |  |  |
