# Supplementary material for: Inactivation times from 290 to 315 nm UVB in sunlight for SARS coronaviruses CoV and CoV-2 using OMI satellite data for the sunlit Earth
Source: Air Qual Atmos Health. 2020 Sep 15;14(2):217–33. doi: 10.1007/s11869-020-00927-2 (PMC7490326; doi:10.1007/s11869-020-00927-2)
Supplement: Supplementary file 3 — (DOCX 30 kb) [file 11869_2020_927_MOESM3_ESM.docx]

Online Resource 3

Table S1: The results (columns 5 – 7) apply to the D_90_ = 40 J/m^2^ SARS CoV and D_90_ = 3.2 J/m^2^ SARS CoV-2 models. Columns 2, 3, and 4 contain each site’s latitude and longitude (degrees), the altitude of each site (km). Columns 5 to 7 contain the smallest value of the T_90_ monthly averages <T_90_> (minutes) (_Min_<T_90_>_12_), and the number N_m_ of such monthly averages (1 to 12) where T_90_ ≤ 120 minutes. For the calculated RS SARS CoV-2 D_90_ = 3.2 J/m^2^, reduce column 5 by a factor of 12.5 to obtain column 7. As shown in Fig. 15, most of the sites have the number of months > 9 for T_90_ ≤ 120 minutes (column 8). It should be noted that the nominal value D_90_ = 40 J/m^2^ is based on laboratory results having a wide range of values (Table 1 items 1 to 6) mostly for different viruses suspended in a liquid medium and measured under a variety of laboratory conditions. This is not the case for the SARS CoV-2 virus responsible for COVID-19, where the inactivation was measured (RS) on a surface exposed to closely simulated sunlight and should represent conditions outdoors in natural sunlight.

| Table S1 Monthly average <T_90_> minimum (minutes) and number of months <T_90_> ≤ 120 minutes | | | | | | | |
| --- | --- | --- | --- | --- | --- | --- | --- |
|  |  |  |  | D_90_=40J/m^2^ | D_90_=40J/m^2^ | D_90_=3.2J/m^2^ | D_90_=3.2J/m^2^ |
| Location | Latitude | Longitude | Elevation | _Min_<T_90_>_12_ | N_m_ | _Min_<T_90_>_12_ | N_m_ |
| Abidjan_CL | 5.34 | -4.02 | 0.01 | 49.58 | 12 | 3.97 | 12 |
| Abuja_HG | 9.07 | 7.49 | 0.01 | 45.41 | 12 | 3.63 | 12 |
| Abu_Dhabi_AE | 24.45 | 54.37 | 0.01 | 49.79 | 10 | 3.98 | 12 |
| Accra_GH | 5.56 | -0.19 | 0.03 | 50.83 | 12 | 4.07 | 12 |
| Adelaide_AU | -34.92 | 138.6 | 0 | 52.6 | 6 | 4.21 | 12 |
| Ahmedabad_IN | 23.03 | 72.59 | 0.06 | 49.28 | 10 | 3.94 | 12 |
| Albuquerque_NM | 35.1 | -106.6 | 1.58 | 59.55 | 6 | 4.76 | 12 |
| Alexandria_EG | 31.21 | 29.92 | 0 | 55.15 | 7 | 4.41 | 12 |
| Algiers_DZ | 36.74 | 3.09 | 0.19 | 59.74 | 5 | 4.78 | 12 |
| Alice_Springs_AU | -23.7 | 133.88 | 0.58 | 38.18 | 10 | 3.05 | 12 |
| Alta_Floresta_BR | 9.87 | -55.64 | 2.02 | 38.91 | 12 | 3.11 | 12 |
| Anchorage_AK_US | 61.1 | -149.9 | 0.03 | 223.21 | 0 | 17.86 | 6 |
| Ankara_TR | 39.93 | 32.87 | 0.85 | 67 | 5 | 5.36 | 12 |
| Annopolis_MD_US | 39.04 | -76.26 | 0 | 86.64 | 4 | 6.93 | 12 |
| Aosta_IT | 45.73 | 7.31 | 0.58 | 106.12 | 2 | 8.49 | 10 |
| Arica_CL | 18.05 | -70.22 | 0.35 | 54.81 | 12 | 4.39 | 12 |
| Athens_GR | 37.98 | 23.73 | 0.72 | 66.01 | 6 | 5.28 | 12 |
| Atlanta_GA_US | 33.5 | -84.5 | 0.31 | 79.92 | 6 | 6.39 | 12 |
| Auckland_NZ | -36.85 | 174.76 | 0.05 | 60.35 | 5 | 4.83 | 12 |
| Baghdad_IQ | 33.34 | 44.4 | 0.04 | 53.73 | 6 | 4.3 | 12 |
| Baltimore_US | 39.3 | -76.61 | 0.05 | 86.86 | 3 | 6.95 | 12 |
| Bangalore_IN | 12.97 | 77.58 | 0.91 | 49.27 | 12 | 3.94 | 12 |
| Bangkok_TH | 13.74 | 100.52 | 0.01 | 48.83 | 12 | 3.91 | 12 |
| Bangor_ME | 44.81 | -68.8 | 0.05 | 114.31 | 2 | 9.14 | 10 |
| Baoding_CN | 38.85 | 115.49 | 0.02 | 81.02 | 3 | 6.48 | 12 |
| Baton_Rouge_US | 30.47 | -91.15 | 0 | 65.93 | 7 | 5.27 | 12 |
| Beijing_CN | 39.9 | 116.4 | 0.05 | 96.34 | 2 | 7.71 | 12 |
| Belsk_PO | 51.96 | 20.3 | 0.17 | 133.53 | 0 | 10.68 | 8 |
| Beltsville_MS_US | 39.03 | -76.76 | 0.03 | 86.49 | 4 | 6.92 | 12 |
| Berlin_DE | 52.52 | 13.41 | 0.04 | 166.52 | 0 | 13.32 | 8 |
| Bogota_CO | 4.62 | -74.06 | 2.54 | 50.08 | 12 | 4.01 | 12 |
| Boston_MA_US | 42.36 | -71.05 | 0.04 | 102.87 | 2 | 8.23 | 10 |
| Brasilia_BR | -15.83 | -47.93 | 1.17 | 50.36 | 12 | 4.03 | 12 |
| Brisbane_AU | -27.47 | 153.03 | 0.03 | 47.5 | 8 | 3.8 | 12 |
| Brussels_BE | 50.8 | 4.35 | 0.1 | 115.04 | 1 | 9.2 | 8 |
| Budapest_HU | 47.93 | 20.5 | 0.29 | 117.69 | 1 | 9.41 | 9 |
| Buenos_Aires_AR | -34.6 | -58.4 | 0.03 | 52.85 | 6 | 4.23 | 12 |
| Bulawayo_ZW | -20.15 | 28.58 | 1.35 | 42.96 | 12 | 3.44 | 12 |
| Busan_KR | 35.17 | 129.07 | 0 | 87.76 | 5 | 7.02 | 12 |
| Cabauw_NL | 51.82 | 4.59 | 0 | 125.16 | 0 | 10.01 | 8 |
| Cairo_EG | 30.06 | 31.25 | 0.02 | 54.04 | 7 | 4.32 | 12 |
| Calgary_CA | 51.05 | -114.08 | 1.04 | 124.01 | 0 | 9.92 | 8 |
| Canberra_AU | -35.28 | 149.13 | 0.57 | 54.84 | 5 | 4.39 | 12 |
| Cape_Town_ZA | -39.92 | 18.42 | 0.04 | 71.36 | 6 | 5.71 | 12 |
| Caracas_VZ | 10.5 | -66.92 | 0.9 | 41.53 | 12 | 3.32 | 12 |
| Casablanca_MA | 33.59 | -7.6 | 0.03 | 57.52 | 6 | 4.6 | 12 |
| Chengdu_CN | 30.67 | 104.07 | 0.5 | 73.93 | 4 | 5.91 | 12 |
| Chennai_IN | 13.07 | 80.24 | 0.02 | 48.25 | 12 | 3.86 | 12 |
| Chicago_IL_US | 41.89 | -87.67 | 0.19 | 84.71 | 4 | 6.78 | 10 |
| Chongqing_CN | 29.56 | 106.55 | 0.22 | 74 | 5 | 5.92 | 12 |
| Christchurch_NZ | -43.53 | 172.64 | 0.02 | 62.2 | 4 | 4.98 | 10 |
| Cordoba_AR | -31.41 | 64.18 | 0.4 | 56.16 | 7 | 4.49 | 12 |
| Dallas_TX_US | 32.78 | -96.81 | 0.14 | 66.8 | 6 | 5.34 | 12 |
| Darwin_AU | -12.5 | 130.8 | 0.01 | 41.56 | 12 | 3.32 | 12 |
| Dar_es_Salaam_TZ | -6.8 | 39.28 | 0.02 | 42.48 | 12 | 3.4 | 12 |
| Delhi_IN | 28.65 | 77.23 | 0.23 | 58.39 | 8 | 4.67 | 12 |
| Denver_CO_US | 39.74 | -104.96 | 1.64 | 66.94 | 5 | 5.35 | 12 |
| Des Moines_IA_US | 41.6 | -93.6 | 0.29 | 73.18 | 4 | 5.85 | 10 |
| Detroit_MI_US | 42.3 | -83.05 | 0.19 | 86.69 | 3 | 6.94 | 10 |
| Dhaka_BD | 23.7 | 90.4 | 0.01 | 57.47 | 10 | 4.6 | 12 |
| Dongguan_CN | 23.02 | 113.75 | 0.01 | 70.09 | 8 | 5.61 | 12 |
| Dubai_AE | 25.07 | 55.17 | 0 | 49.74 | 9 | 3.98 | 12 |
| Eureka_CA_US | 40.8 | -124.1 | 0.01 | 76.07 | 4 | 6.09 | 11 |
| Flagstaff_AZ_US | 35.2 | -111.65 | 2.13 | 58.32 | 6 | 4.67 | 12 |
| Giza_EG | 30.01 | 31.21 | 0.03 | 53.99 | 7 | 4.32 | 12 |
| Glascow_UK | 55.91 | -3.97 | 0.16 | 202.29 | 0 | 16.18 | 7 |
| Greenbelt_MD_US | 39 | -76.9 | 0.06 | 86.32 | 4 | 6.91 | 12 |
| Grenada_ES | 37.16 | -3.55 | 0.81 | 61.33 | 6 | 4.91 | 12 |
| Griffin_GA_US | 33.2 | -84.28 | 0.29 | 79.61 | 6 | 6.37 | 12 |
| Guangzhou_CN | 23.13 | 113.25 | 0.01 | 70.11 | 8 | 5.61 | 12 |
| Hamilton_NZ | -37.85 | 175.32 | 0.05 | 59.53 | 5 | 4.76 | 12 |
| Hangzhou_CN | 30.29 | 120.16 | 0.01 | 74.36 | 5 | 5.95 | 12 |
| Hanoi_VN | 21.02 | 105.84 | 0.01 | 58.86 | 8 | 4.71 | 12 |
| Hartford_CT_US | 41.8 | -72.8 | 0.01 | 95.5 | 3 | 7.64 | 10 |
| Havana_CU | 23.25 | -82.66 | 0 | 52.86 | 9 | 4.23 | 12 |
| Helsinki_FI | 61.92 | 25.75 | 0.01 | 202.9 | 0 | 16.23 | 6 |
| Hong_Kong_CN | 22.29 | 114.16 | 0 | 56.42 | 9 | 4.51 | 12 |
| Honolulu_HI_US | 21.3 | -157.8 | 0.01 | 49.62 | 10 | 3.97 | 12 |
| Houston_TX_US | 29.75 | -95.36 | 0.01 | 63.47 | 7 | 5.08 | 12 |
| Ho_Chi_Minh_VN | 10.76 | 106.66 | 0.01 | 46.63 | 12 | 3.73 | 12 |
| Hyderabad_IN | 17.36 | 78.46 | 0.51 | 51.71 | 12 | 4.14 | 12 |
| Indianapolis_OH_US | 39.77 | -86.16 | 0.25 | 91.62 | 3 | 7.33 | 11 |
| Iowa_Center_IA_US | 42 | -93.5 | 0.3 | 78.56 | 3 | 6.28 | 10 |
| Iquitos_PE | -3.75 | -73.25 | 0.13 | 56.25 | 12 | 4.5 | 12 |
| Ispra_IT | 45.82 | 7.72 | 2.01 | 99.44 | 4 | 7.96 | 10 |
| Istanbul_CN | 41.02 | 28.98 | 0.04 | 80.92 | 4 | 6.47 | 11 |
| Izania_ES | 28.33 | -16.56 | 1.19 | 56.75 | 7 | 4.54 | 12 |
| Jakarta_ID | -6.21 | 106.85 | 0.08 | 57.17 | 12 | 4.57 | 12 |
| Jokioinen_FI | 60.8 | 23.48 | 0.12 | 204.75 | 0 | 16.38 | 6 |
| Kansas_City_US | 39.09 | -94.57 | 0.29 | 67.8 | 5 | 5.42 | 12 |
| Karachi_PK | 24.95 | 67.01 | 0 | 51.63 | 10 | 4.13 | 12 |
| Kinshasa_CD | -4.32 | 15.31 | 0.31 | 47.55 | 12 | 3.8 | 12 |
| Kislovodsk_RU | 43.91 | 42.72 | 0.81 | 93.54 | 1 | 7.48 | 10 |
| Lagos_NG | 6.47 | 3.41 | 0.01 | 46.55 | 12 | 3.72 | 12 |
| Lahore_PK | 31.55 | 74.34 | 0.22 | 53.16 | 8 | 4.25 | 12 |
| Lamar_CO_US | 38.09 | -102.62 | 1.1 | 63.47 | 6 | 5.08 | 12 |
| Lansing_MI_US | 42.73 | -84.56 | 0.03 | 95.54 | 3 | 7.64 | 10 |
| Lauder_NZ | -45.05 | 169.7 | 0.37 | 82 | 2 | 6.56 | 10 |
| La_Paz_BO | -16.5 | -68.15 | 3.78 | 37.61 | 12 | 3.01 | 12 |
| La_Quiaca_AR | -22.11 | -65.57 | 4.46 | 40.06 | 12 | 3.2 | 12 |
| Leeds_UK | 53.8 | -1.55 | 0.03 | 190.01 | 0 | 15.2 | 7 |
| Lima_PE | -12.04 | -77.03 | 0.15 | 39.77 | 10 | 3.18 | 12 |
| London_UK | 51.51 | -0.12 | 0.02 | 145.9 | 0 | 11.67 | 8 |
| Los_Angeles_CA_US | 34.5 | -118.5 | 0.1 | 57.54 | 6 | 4.6 | 12 |
| Madrid_ES | 40.42 | -3.7 | 0.67 | 66.12 | 5 | 5.29 | 12 |
| Makassar_ID | -5.13 | 119.4 | 0.01 | 42.95 | 12 | 3.44 | 12 |
| Manaus_BR | -3.12 | -60 | 0.09 | 53.04 | 12 | 4.24 | 12 |
| Manchester_UK | 53.6 | -1.97 | 0.33 | 185.78 | 0 | 14.86 | 8 |
| Manhattan_NY_US | 40.76 | -73.97 | 0.01 | 83.24 | 3 | 6.66 | 12 |
| Marimbo_AR | -33.42 | -63.29 | 0.2 | 52.12 | 7 | 4.17 | 12 |
| Marin_County_CA_US | 37.5 | -122 | 0.1 | 63.25 | 5 | 5.06 | 12 |
| Mauna_Loa_Obs_HI | 19.54 | 155.6 | 3.4 | 43.95 | 12 | 3.52 | 12 |
| Melbourne_AU | -37.3 | 145 | 0.01 | 56.6 | 5 | 4.53 | 12 |
| Mendoza_AR | -32.9 | -68.9 | 0.83 | 45.38 | 7 | 3.63 | 12 |
| Mexico_City_MX | 19.43 | -99.13 | 2.24 | 48.1 | 12 | 3.85 | 12 |
| Miami_FL_US | 25.77 | -80.19 | 0.03 | 63.68 | 8 | 5.09 | 12 |
| Monterrey_MX | 25.68 | -100.32 | 1.78 | 50.59 | 7 | 4.05 | 12 |
| Montreal_CA | 45.45 | -79.93 | 0.02 | 100.99 | 2 | 8.08 | 9 |
| Moscow_RU | 55.75 | 37.62 | 0.14 | 164.91 | 0 | 13.19 | 8 |
| Mt_Everest_0km | 28 | 86.9 | 0 | 63.54 | 8 | 5.08 | 12 |
| Mt_Everest_8.85 | 28 | 86.9 | 8.85 | 44.05 | 11 | 3.52 | 12 |
| Mt_Kenya_KE | 0.13 | 37.3 | 5.2 | 33.37 | 12 | 2.67 | 12 |
| Mumbai_IN | 19.08 | 72.88 | 0.02 | 48.3 | 12 | 3.86 | 12 |
| NAHA_JP | 26.21 | 127.68 | 0.05 | 59.01 | 8 | 4.72 | 12 |
| Nairobi_KE | 1.09 | 35.88 | 1.86 | 38.74 | 12 | 3.1 | 12 |
| Nanjing_CN | 32.06 | 118.78 | 0.02 | 75.71 | 5 | 6.06 | 12 |
| New_Delhi_IN | 28.61 | 77.2 | 0.03 | 58.95 | 8 | 4.72 | 12 |
| New_Orleans_US | 29.95 | -90.08 | 0.02 | 64.08 | 7 | 5.13 | 12 |
| New_York_US | 40.71 | -71.01 | 0.06 | 83.69 | 3 | 6.7 | 11 |
| Nice_FR | 43.67 | 7.29 | 0.03 | 89.96 | 4 | 7.2 | 11 |
| Obninsk_RU | 55.1 | 36.61 | 0.17 | 159.03 | 0 | 12.72 | 8 |
| Palembang_ID | -2.99 | 104.76 | 0.01 | 53.25 | 12 | 4.26 | 12 |
| Paris_FR | 48.86 | 2.35 | 0.04 | 97.17 | 1 | 7.77 | 8 |
| Perth_AU | -31.95 | 115.9 | 0.03 | 45.89 | 6 | 3.67 | 12 |
| Phoenix_US | 33.45 | -112.07 | 0.37 | 55.17 | 6 | 4.41 | 12 |
| Pilar_AR | -31.66 | -63.88 | 0.34 | 44.22 | 7 | 3.54 | 12 |
| Portland_US | 45.52 | -122.67 | 0.01 | 89.72 | 2 | 7.18 | 9 |
| Punta_Arenas_CL | -53.16 | -70.92 | 0.04 | 123.46 | 0 | 9.88 | 8 |
| Quanzhou_CN | 24.91 | 116.59 | 0.01 | 70.01 | 6 | 5.6 | 12 |
| Queenstown_SA | -31.9 | 26.92 | 1.1 | 45.06 | 8 | 3.61 | 12 |
| Quezon_City_PH | 14.65 | 121.05 | 0.05 | 47.91 | 11 | 3.83 | 12 |
| Quito_EC | 0.18 | -78.5 | 2.85 | 65.04 | 12 | 5.2 | 12 |
| Recife_BR | -8.05 | -34.93 | 0.55 | 40.78 | 12 | 3.26 | 12 |
| Redding_CA_US | 40.5 | -122.4 | 0.03 | 69.26 | 4 | 5.54 | 11 |
| Rio_de_Janeiro_BR | -22.91 | -43.2 | 0.05 | 59.94 | 9 | 4.8 | 12 |
| Riyadh_SA | 24.77 | 46.74 | 0.61 | 46.63 | 10 | 3.73 | 12 |
| Rome_IT | 41.9 | 12.5 | 0.01 | 74.05 | 4 | 5.92 | 11 |
| Rosario_AR | -32.94 | -60.64 | 0.03 | 53.45 | 6 | 4.28 | 12 |
| Rural_Georgia_GA | 34.5 | -83.5 | 0.2 | 84.62 | 6 | 6.77 | 12 |
| Sacramento_CA_US | 38.5 | -121.5 | 0.08 | 65.87 | 6 | 5.27 | 12 |
| Saint_Petersburg_RU | 59.98 | 30.32 | 0.01 | 195.06 | 0 | 15.6 | 6 |
| Salt_Lake_UT_US | 40.7 | -111.9 | 1.32 | 65.1 | 5 | 5.21 | 11 |
| Salvador_BR | -12.97 | -38.48 | 0.01 | 49.03 | 12 | 3.92 | 12 |
| San Diego_CA_US | 32.77 | 117.19 | 0.01 | 72.93 | 5 | 5.83 | 12 |
| Santa FE_NM_US | 35.69 | -105.94 | 2.14 | 57 | 6 | 4.56 | 12 |
| Santa_Rosa_CA_US | 38.5 | -122.7 | 0.05 | 63.66 | 6 | 5.09 | 12 |
| Santiago_CL | -33.46 | -70.65 | 0.56 | 41.15 | 5 | 3.29 | 12 |
| San_Antonio_TX_US | 29.42 | -98.49 | 0.2 | 62.85 | 6 | 5.03 | 12 |
| San_Francisco_US | 37.77 | -122.42 | 0.03 | 72.94 | 5 | 5.84 | 12 |
| San_Jose_CA_US | 37.5 | -122.5 | 0.14 | 72.2 | 5 | 5.78 | 12 |
| San_Julian_AR | -49.32 | -67.75 | 0.06 | 85.57 | 3 | 6.85 | 9 |
| San_Pedro_CL | -22.9 | -68.2 | 2.45 | 34.66 | 11 | 2.77 | 12 |
| Sao Paulo_BR | -23.55 | -46.64 | 0.77 | 55.25 | 8 | 4.42 | 12 |
| Sapporo_JP | 43.08 | 140.76 | 0.36 | 95.23 | 2 | 7.62 | 9 |
| Seattle_WA_US | 47.5 | -123.5 | 0.14 | 103.88 | 2 | 8.31 | 9 |
| Seoul_KR | 37.56 | 126.98 | 0.04 | 86.41 | 4 | 6.91 | 12 |
| Shanghai_CN | 31.22 | 121.47 | 0.06 | 79.69 | 6 | 6.38 | 12 |
| Shenyang_CN | 41.79 | 123.43 | 0.05 | 109.38 | 2 | 8.75 | 10 |
| Shenzhen_CN | 22.55 | 114.07 | 0 | 56.44 | 9 | 4.51 | 12 |
| Singapore_SG | 1.29 | 103.85 | 0.02 | 48.53 | 12 | 3.88 | 12 |
| Sodankylä_FI | 67.42 | 26.6 | 0.18 | 194.55 | 0 | 15.56 | 4 |
| Stanley_FK | -51.7 | -57.9 | 0.05 | 123.04 | 0 | 9.84 | 8 |
| Steamboat_Spr_US | 40.48 | -106.83 | 2.07 | 63.8 | 5 | 5.1 | 12 |
| St_Louis_MO_US | 38.63 | -90.2 | 0.15 | 78.81 | 5 | 6.31 | 12 |
| Suzhou_CN | 31.3 | 120.6 | 0.01 | 71.27 | 5 | 5.7 | 12 |
| Tampa_FL_US | 28 | -82.5 | 0.01 | 67.21 | 7 | 5.38 | 12 |
| Tehran_IR | 35.69 | 51.42 | 1.18 | 52.08 | 6 | 4.17 | 12 |
| Tel-Aviv_IL | 32.11 | 34.86 | 0.03 | 55.86 | 7 | 4.47 | 12 |
| Tianjin_CN | 39.14 | 117.18 | 0.01 | 91.56 | 3 | 7.32 | 12 |
| Tokyo_JP | 35.65 | 139.84 | 0.04 | 86.44 | 6 | 6.92 | 12 |
| Toronto_CA | 43.65 | -79.35 | 0.17 | 96.18 | 2 | 7.69 | 10 |
| Tuscon_AZ_US | 32.22 | -110.3 | 0.76 | 57.64 | 6 | 4.61 | 12 |
| Ushuaia_AR | -54.8 | -68.3 | 0.06 | 139.77 | 0 | 11.18 | 8 |
| Utah_Center_UT_US | 39 | -109.5 | 1.8 | 63.75 | 5 | 5.1 | 12 |
| Vancouver_CA | 49.24 | -123.12 | 0.07 | 117.85 | 1 | 9.43 | 9 |
| Vientiane_LA | 17.97 | 102.63 | 0.17 | 57.09 | 12 | 4.57 | 12 |
| Waimea_HA_US | 21.96 | -159.68 | 0.01 | 49.74 | 10 | 3.98 | 12 |
| Washington_DC_US | 38.9 | -77.04 | 0.01 | 82.73 | 4 | 6.62 | 12 |
| Wellington_NZ | -41.3 | 174.8 | 0.08 | 65.02 | 5 | 5.2 | 11 |
| Wenzhou_CN | 28 | 120.67 | 0.01 | 59.78 | 5 | 4.78 | 12 |
| White_Sands_NM | 32.4 | -106.5 | 1.22 | 53.91 | 7 | 4.31 | 12 |
| Wuhan_CN | 30.58 | 114.27 | 0.02 | 68.26 | 6 | 5.46 | 12 |
| Yangon_MM | 30.58 | 114.27 | 0.02 | 68.26 | 6 | 5.46 | 12 |
| Zugspitze_DE | 30.58 | 114.27 | 0.02 | 68.26 | 6 | 5.46 | 12 |

* The height dependence calculation for Mt. Everest was extrapolated to 8.85 km from 8 km.

The effect of altitude on <T_90_> is clearly shown by artificially setting the height of Mt Everest to 0 km. The result is that for D_90_=40J/m^2^ minimum <T_90_> increases from 44 minutes to 64 minutes and the number of months <T_90_> < 120 minutes decreases from 11 to 8 months.
